# The Relative Contribution of Plasma Homocysteine Levels vs. Traditional Risk Factors to the First Stroke: A Nested Case-Control Study in Rural China

**DOI:** 10.3389/fmed.2021.727418

**Published:** 2022-01-20

**Authors:** Feng Zhou, Chengzhang Liu, Lijing Ye, Yukai Wang, Yan Shao, Guohua Zhang, Zhenpeng Duan, Jingjuan Chen, Jingyun Kuang, Jingyi Li, Yun Song, Lishun Liu, Pierre Zalloua, Xiaobin Wang, Xiping Xu, Chengguo Zhang

**Affiliations:** ^1^Department of Neurology, First People's Hospital of Foshan, Foshan, China; ^2^Research Center of Clinical Medicine, Nanfang Hospital, Southern Medical University, Guangzhou, China; ^3^Shenzhen Evergreen Medical Institute, Shenzhen, China; ^4^Department of Epidemiology and Biostatistics, School of Public Health, Anhui Medical University, Hefei, China; ^5^Institute of Biomedicine, Anhui Medical University, Hefei, China; ^6^State Key Laboratory of Natural Medicines, Research Center of Biostatistics and Computational Pharmacy, China Pharmaceutical University, Nanjing, China; ^7^Beijing Advanced Innovation Center for Food Nutrition and Human Health, College of Food Science and Nutritional Engineering, China Agricultural University, Beijing, China; ^8^School of Medicine, Lebanese American University, Byblos, Lebanon; ^9^Department of Population, Family and Reproductive Health, Johns Hopkins University Bloomberg School of Public Health, Baltimore, MD, United States; ^10^The State Key Laboratory for Organ Failure Research, National Clinical Research Study Center for Kidney Disease, Guangzhou, China; ^11^Renal Division, Nanfang Hospital, Southern Medical University, Guangzhou, China

**Keywords:** homocysteine, systolic blood pressure, first stroke, ischemic stroke, population attributable risk

## Abstract

**Background:**

Approximately 75% of Chinese hypertensive patients have elevated homocysteine (Hcy). Its implication in risk assessment and prevention of the first stroke remains an important clinical and public health question.

**Methods:**

This study was based on a community cohort recruited from 2016 to 2018 in the rural China. To maximize cost efficiency, we used a nested case-control design, including 3,533 first stroke cases and 3,533 controls matched for age ±1 years, sex, and village. Individual associations of tHcy and traditional risk factors with the first stroke were examined, and their population-attributable risks (PARs) were estimated.

**Results:**

There was a significant dose-response association between first stroke and total Hcy (tHcy) levels, with adjusted odds ratios of 1.11 (95% CI: 0.97, 1.26) for tHcy 10–15 μmol/L and 1.44 (1.22, 1.69) for tHcy ≥ 15 μmol/L, all compared to tHcy < 10 μmol/L. A similar trend was found for ischemic and hemorrhagic stroke. tHcy and systolic blood pressure (SBP) were independently and additively associated with the risk of first stroke (tHcy: 1.06 [1.02, 1.1]; SBP: 1.13 [1.1, 1.16]; *P*-interaction, 0.889). Among the ten main risk factors examined, the top two contributors to the first stroke were SBP and tHcy, with PARs of 25.73 and 11.24%, respectively.

**Conclusions:**

Elevated tHcy is the second most important contributor and acts additively with SBP to increase the risk of the first stroke. This finding underscores the importance of screening and treating elevated tHcy along with traditional risk factors to further reduce the burden of the first stroke in the high-risk populations.

## Introduction

Stroke is the second leading cause of death and disability worldwide and the leading cause of death in China (1). China has the highest stroke burden in the world (2), and this burden has been increasing over the past 4 decades, particularly in the rural areas (3, 4). While stroke in China share traditional risk factors, the high and escalating stroke rate urges us to investigate distinctive feature of stroke epidemiology in China and identify additional intervention targets (5).

Hypertension is the most important modifiable risk factor for stroke risk (6). In addition, a unique and well-observed clinical feature is that approximately 75% of Chinese hypertensive patients have hyperhomocysteinemia (HHcy) (7). The high prevalence of HHcy in China is due to several reasons. Unlike the US, China is a country without mandatory folic acid fortification. The Chinese diet plus cooking methods result in a low intake of folate and folic acid. Chinese population has a high rate of C677T mutation in the methylenetetrahydrofolate reductase (*MTHFR)* gene encoding a homocysteine (Hcy) metabolism-related enzyme (8). Although the independent and interactive impacts of HHcy and hypertension on cardiovascular diseases have been previously reported (9, 10), few recent studies have been conducted to quantify the relative effect of HHcy, in the context of traditional risk factors, on the first stroke, ischemic stroke, and hemorrhagic stroke, especially among rural Chinese community populations who have a disproportionately high burden of stroke and its severe health and economic sequala.

Population-attributable risk (PAR) provides insight into the relative significance of a given risk factor on stroke risk in the general population and can be used to predict the impact of public health interventions on adverse outcomes (11). To date, the PARs of tHcy and potentially modifiable etiological factors for the first stroke in the rural Chinese population remain mostly unknown.

Therefore, we conducted this nested case-control study, estimated to what extent HHcy alone and in conjunction with systolic blood pressure (SBP) can contribute to the risk of the first stroke. We also computed PARs for HHcy and other individual risk factors to estimate the proportion of the first stroke that could be prevented by the elimination of etiological factors from the population.

## Methods

### Study Design and Population

Our present study was a subset of the “H-type Hypertension and Stroke Prevention and Control Project”, which is a community-based, observational, multicenter, real-world registry study, and was conducted in the rural areas of Rongcheng County, and Lianyungang County, China. The detailed inclusion and exclusion criteria, follow-up, and outcomes of the study have been described in a previous publication (12). Briefly, eligible participants were local residents aged ≥ 35 years with essential hypertension, defined as a mean seated SBP ≥ 140 mmHg or diastolic blood pressure (DBP) ≥ 90 mmHg at the screening visit. The exclusion criteria were as follows: confirmed stroke at the time of screening; diagnosed secondary hypertension; and a history of cancer, myocardial infarction, or severe mental diseases. In the first stage, participants were screened and recruited, and the baseline data were collected; in the second stage of the 3-year observation, they were scheduled for follow-up every 3 months.

**Figure 1:**
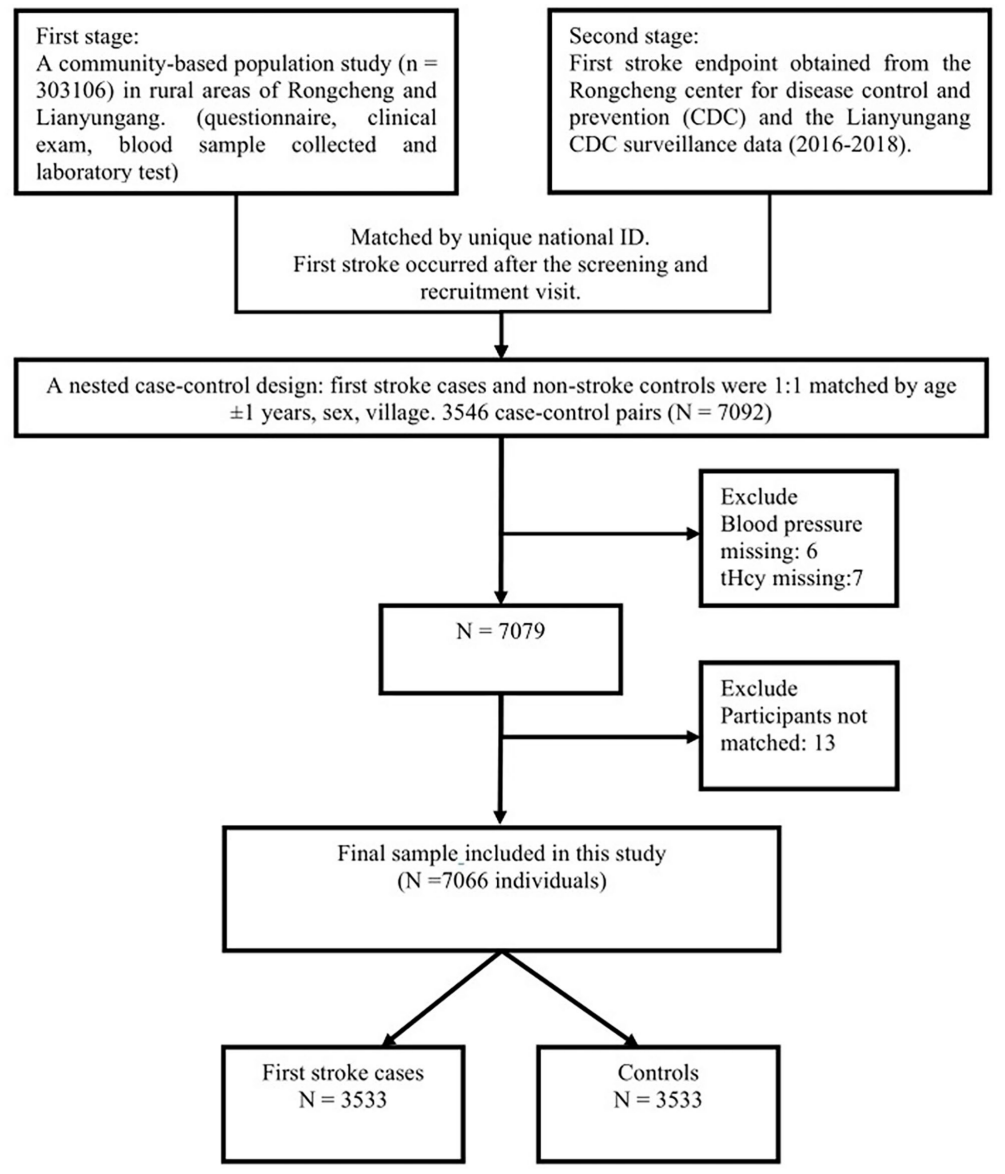
Flow chart of the study design and participants. A total of 3,533 cases were individually matched to 3,533 controls by age ±1 years, sex, and village.

We implemented this nested case-control study design because it was economical and extrapolates the data well and matching lowers the interference of confounding factors to a certain degree. In the second stage, patients with stroke data from the Rongcheng Center for Disease Control and Prevention (CDC) and the Lianyungang CDC who had complete records that were selected as cases. First stroke cases and nonstroke controls were 1:1 matched by age ±1 years, sex, and village. The initial sample consisted of 3,546 pairs. Next, we excluded the participants with missing values of blood pressure (*n* = 6) and tHcy (*n* = 7) and unpaired individuals (*n* = 13). Based on the inclusion and exclusion criteria, 3,533 stroke cases and 3,533 matched controls with complete Hcy measurements were selected for the final data analysis. The detailed procedure is presented in Figure 1.

### Baseline Assessments and Definitions

Baseline data collection was conducted in the first stage by trained research staff using uniform standard operating procedures. Questionnaires were administered to collect information on demographics, life habits, and the physical intensity of the job. Body mass index (BMI) was calculated as the bodyweight divided by the square of the height (kg/m^2^). The participants were classified as never, former, or current smokers *via* a self-reported survey. The physical intensity of the job was measured *via* a validated scale and classified as low, moderate, or high (13). Clinical and medical information was obtained from medical records. Diabetes was defined as fasting plasma glucose ≥ 7 mmol/L or taking diabetes medications. Coronary heart disease (CHD) was defined as previously documented myocardial infarction, coronary revascularization, or patients with symptoms of electrocardiographic modifications (14).

Venous blood was drawn after overnight fasting during the baseline visit. Plasma and serum samples were collected and subsequently stored at −80°C until laboratory tests were conducted. Plasma tHcy, fasting glucose, creatinine, triglycerides (TG), total cholesterol (TC), and high-density lipoprotein (HDL) were measured by the automated analyzer. Low-density lipoprotein (LDL) was calculated by the Friedewald formula (15). The estimated glomerular filtration rate (eGFR) was calculated using the Chronic Kidney Disease Epidemiology Collaboration equation (16). HHcy was defined in various ways: (1) plasma total homocysteine (tHcy) levels ≥ 15 μmol/L by American guidelines and subsequently adjusted to ≥ 10 μmol/L by the American Heart Association/American Stroke Association guidelines on the primary prevention of stroke (17, 18), (2) > 12 μmol/L by German, Austrian and Swiss Homocysteine Society guidelines (19), and (3) ≥ 10 μmol/L by the Chinese guidelines (20). In this study, we defined the categories of tHcy as <10, 10 to <15, and ≥ 15 μmol/L.

### Outcomes

The primary outcome of interest was a first, nonfatal or fatal symptomatic stroke (ischemic and hemorrhagic). Silent stroke and subarachnoid hemorrhage were excluded. The first stroke was confirmed by the CT and/or MRI and was diagnosed based on the presence of the International Classification of Diseases (ICD)-10 codes. Secondary outcomes included ischemic stroke and hemorrhagic stroke. Using a standardized form, we collected data from eligible consenting first-stroke patients who were enrolled in the CDC surveillance.

### Statistical Analysis

All of the analyses were conducted using R software (version 3.5.3; http://www.R-project.org) and Empower (version 2.17.9; www.empowerstats.com). A two-tailed *P* < 0.05 was considered to be statistical significant in all analyses.

Baseline characteristics are presented as the mean (SD) for continuous variables and as proportions (%) for categorical variables. Statistical differences between cases and controls were calculated by paired *t*-test for continuous variables and by chi-squared tests for the categorical variables. Conditional logistic regression analysis was performed to assess the odds ratio (OR) and 95% CI for the association between HHcy, traditional risk factors, and the risk of the first stroke, ischemic and hemorrhagic strokes, other than for stratified analyses, for which we used unconditional logistic regression. We used smoothing curve fitting to further characterize the shape of the relationship between tHcy, SBP, and first stroke and its subtypes. All the regression analyses were adjusted for pertinent covariates. The categories of covariates are as follows: center (Rongcheng, Lianyungang), age (< 65, ≥ 65 years), sex (male, female), BMI (< 28, ≥ 28 kg/m^2^), smoking status (never, former, and current), physical intensity of job (low, moderate, and high), tHcy (< 10, 10 to < 15, ≥ 15 μmol/L), SBP (< 140, 140 to < 160, ≥ 160 mmHg), intake of antihypertensive drugs (no, yes), diabetes (no, yes), CHD (no, yes), TC (< 5.2, ≥ 5.2 mmol/L), TG (< 1.7, ≥ 1.7 mmol/L), HDL (male ≥ 1.03/female ≥ 1.3, male < 1.03/female < 1.3 mmol/L), LDL (< 3.4, ≥ 3.4 mmol/L), eGFR (≥ 90, < 90 ml/min/1.732 m^2^).

Adjusted PARs with respective 95% CIs for individual risk factors and their combinations for the first stroke and its subtypes were calculated by logistic regression models and adjusted for confounding. The method was introduced in study of Bruzzi (21). The PAR point estimator is implemented in the R package attribrisk.

## Results

### Baseline Characteristics

Among 3,533 first stroke cases, 3,070 (86.9%) had an ischemic stroke, 426 (12.1%) had a hemorrhagic stroke, and 37 (1.0%) had mixed lesions of both ischemia and hemorrhage. The mean age at blood sample collection was 67.9 (SD, 9.3) years for first stroke cases and 67.8 (SD, 9.3) years for controls; females made up 55.3%. Compared with controls, cases tended to have a higher BMI; higher levels of SBP, DBP, fasting glucose, tHcy, and TG; and lower levels of eGFR at baseline (*P* < 0.05). In addition, cases had higher proportions of medication use than controls (*P* < 0.05). Cases with mixed strokes were excluded from stroke subtype analyses. Similar trends were found in ischemic and hemorrhagic strokes (Table 1).

**Table 1:** Baseline characteristics of the study participants.

	**First stroke**		**Ischemic stroke**		**Hemorrhagic stroke**		**Mixed stroke**	
	**Controls**	**Cases**	**Controls**	**Cases**	**Controls**	**Cases**	**Controls**	**Cases**
*N*	3,533	3,533	3,070	3,070	426	426	37	37
Age, years	67.8 (9.3)	67.9 (9.3)	68.0 (9.2)	68.0 (9.2)	66.7 (10.0)	66.8 (10.0)	66.1 (8.7)	66.1 (8.8)
Female	1,953 (55.3)	1,953 (55.3)	1,705 (55.5)	1,705 (55.5)	223 (52.3)	223 (52.3)	25 (67.6)	25 (67.6)
Body mass index, kg/m^2^	25.6 (3.7)	26.1 (4.1)	25.6 (3.7)	26.1 (4.1)	25.7 (3.8)	26.1 (4.4)	26.5 (4.9)	25.4 (3.7)
Systolic blood pressure, mmHg	148.2 (20.9)	153.8 (21.9)	148.2 (20.9)	153.2 (21.8)	148.4 (20.9)	157.2 (21.8)	151.2 (20.4)	159.8 (25.6)
Diastolic blood pressure, mmHg	86.5 (12.8)	90.3 (13.7)	86.5 (12.8)	89.8 (13.4)	86.5 (12.3)	93.2 (15.4)	90.5 (12.7)	94.1 (14.6)
Smoking status								
never	2,561 (72.5)	2,514 (71.2)	2,230 (72.6)	2, 191 (71.4)	301 (70.7)	292 (68.5)	30 (81.1)	31 (83.8)
former	286 (8.1)	271 (7.7)	250 (8.1)	221 (7.2)	36 (8.5)	49 (11.5)	0 (0.0)	1 (2.7)
current	686 (19.4)	748 (21.2)	590 (19.2)	658 (21.4)	89 (20.9)	85 (20.0)	7 (18.9)	5 (13.5)
Physical intensity of job								
low	2, 170 (61.4)	2,301 (65.1)	1,894 (61.7)	2,013 (65.6)	260 (61.0)	272 (63.8)	16 (43.2)	16 (43.2)
moderate	989 (28.0)	888 (25.1)	854 (27.8)	758 (24.7)	117 (27.5)	112 (26.3)	18 (48.6)	18 (48.6)
high	374 (10.6)	344 (9.7)	322 (10.5)	299 (9.7)	49 (11.5)	42 (9.9)	3 (8.1)	3 (8.1)
Disease History								
Hypertension	2,798 (79.2)	3, 101 (87.8)	2,430 (79.2)	2,677 (87.2)	335 (78.6)	388 (91.1)	33 (89.2)	36 (97.3)
Coronary heart disease	281 (8.0)	365 (10.3)	239 (7.8)	319 (10.4)	40 (9.4)	40 (9.4)	2 (5.4)	6 (16.2)
Diabetes	625 (17.7)	893 (25.3)	535 (17.4)	810 (26.4)	78 (18.3)	77 (18.1)	12 (32.4)	6 (16.2)
Medication used								
Antihypertensive drugs	1,372 (38.8)	1,744 (49.4)	1,206 (39.3)	1,528 (49.8)	152 (35.7)	207 (48.6)	14 (37.8)	9 (24.3)
Glucose-lowering drugs	174 (4.9)	285 (8.1)	151 (4.9)	273 (8.9)	21 (4.9)	12 (2.8)	2 (5.4)	0 (0.0)
Laboratory tests								
Fasting glucose, mmol/L	6.0 (2.0)	6.5 (2.5)	6.0 (2.0)	6.5 (2.5)	6.0 (1.9)	6.2 (2.3)	7.0 (3.3)	6.7 (4.1)
Homocysteine, μmol/L	13.4 (7.9)	14.1 (8.0)	13.4 (8.2)	14.1 (7.8)	13.1 (6.0)	14.4 (9.4)	13.9 (7.1)	12.6 (4.9)
Total cholesterol, mmol/L	5.5 (1.2)	5.5 (1.2)	5.5 (1.2)	5.5 (1.2)	5.4 (1.2)	5.6 (1.2)	5.4 (1.0)	5.5 (1.4)
Triglyceride, mmol/L	1.4 (0.9)	1.6 (1.0)	1.4 (1.0)	1.6 (1.1)	1.4 (0.9)	1.5 (0.9)	1.5 (0.8)	1.7 (0.9)
High-density lipoprotein, mmol/L	1.6 (0.4)	1.6 (0.4)	1.6 (0.4)	1.5 (0.4)	1.6 (0.4)	1.6 (0.4)	1.6 (0.4)	1.6 (0.4)
Low-density lipoprotein, mmol/L	3.3 (0.8)	3.3 (0.9)	3.3 (0.8)	3.3 (0.9)	3.2 (0.9)	3.3 (0.8)	3.1 (0.7)	3.2 (0.9)
eGFR, ml/min/1.73 m^2^	94.9 (13.7)	93.4 (15.1)	94.6 (13.6)	93.3 (14.9)	96.5 (13.8)	94.3 (16.3)	98.5 (13.3)	96.2 (13.0)

*Continuous variables are presented as mean (SD), categorical variables are presented as n (%)*.

*eGFR, estimated glomerular filtration rate*.

### Effects of tHcy and Traditional Risk Factors on the First Stroke

Overall, there were significant associations between tHcy, traditional risk factors, and first stroke in the general population (Table 2), in sex subgroups (Table A1 in Supplementary Material), in age subgroups (Table A2 in Supplementary Material), and in center subgroups (Table A3 in Supplementary Material). In the general population, tHcy [10–15 vs. < 10 μmol/L, OR: 1.11 (95% CI: 0.97, 1.26); ≥ 15 vs. < 10 μmol/L, 1.44 (1.22, 1.69)], SBP [140–160 vs. < 140 mmHg, 1.38 (1.22, 1.57); ≥ 160 vs. < 140 mmHg, 1.85 (1.60, 2.14)], smoking status [ever vs. never, 1.16 (1.01, 1.34)], diabetes [yes vs. no, 1.44 (1.28, 1.63)], and eGFR [< 90 vs. ≥ 90 ml/min/1.73 m^2^, 1.19 (1.03, 1.36)] were significantly associated with adjusted first stroke risk. Regarding ischemic stroke, tHcy, SBP, smoker, diabetes, and TG were significantly associated with adjusted ischemic stroke risk. Nevertheless, only tHcy and SBP were significantly associated with the adjusted hemorrhagic stroke risk.

**Table 2:** The associations of tHcy and traditional risk factors with the risk of first stroke, ischemic, and hemorrhagic stroke.

	**First stroke (3,533 pairs)**		**Ischemic stroke (3,070 pairs)**		**Hemorrhagic stroke (426 pairs)**	
	**Crude**	**Adjusted**	**Crude**	**Adjusted**	**Crude**	**Adjusted**
tHcy, μmol/L						
<10	ref	ref	Ref	ref	ref	ref
10 <15	1.11 (0.98, 1.26)	1.11 (0.97, 1.26)	1.08 (0.94, 1.24)	1.08 (0.94, 1.25)	1.30 (0.90, 1.89)	1.32 (0.88, 1.98)
≥15	1.53 (1.31, 1.79)	1.44 (1.22, 1.69)	1.46 (1.24, 1.73)	1.39 (1.16, 1.66)	2.11 (1.33,3.34)	1.81 (1.09,3.02)
SBP, mmHg						
<140	ref	ref	Ref	ref	ref	ref
140- <160	1.44 (1.28, 1.63)	1.38 (1.22, 1.57)	1.37 (1.21, 1.57)	1.31 (1.15, 1.51)	2.37 (1.56,3.58)	2.21 (1.44,3.40)
≥160	2.10 (1.82,2.41)	1.85 (1.60,2.14)	1.95 (1.68,2.27)	1.72 (1.47,2.02)	3.79 (2.44,5.91)	3.39 (2.13,5.40)
Body mass index, kg/m^2^						
<28	ref	ref	Ref	ref	ref	ref
≥28	1.18 (1.05, 1.31)	1.01 (0.90, 1.13)	1.19 (1.06, 1.34)	1.02 (0.90, 1.16)	1.19 (0.88, 1.61)	0.95 (0.68, 1.34)
Smoking status						
Never	ref	ref	Ref	ref	ref	ref
Ever	1.12 (0.98, 1.28)	1.16 (1.01, 1.34)	1.11 (0.96, 1.29)	1.17 (1.00, 1.36)	1.17 (0.81, 1.68)	1.01 (0.67, 1.51)
Diabetes						
No	ref	ref	Ref	ref	ref	ref
Yes	1.57 (1.40, 1.76)	1.44 (1.28, 1.63)	1.70 (1.50, 1.93)	1.56 (1.37, 1.78)	0.98 (0.70, 1.39)	0.96 (0.66, 1.41)
Total cholesterol, mmol/L						
<5.2	ref	ref	Ref	ref	ref	ref
≥5.2	1.00 (0.91, 1.11)	0.99 (0.88, 1.10)	0.97 (0.87, 1.08)	0.96 (0.85, 1.08)	1.26 (0.94, 1.69)	1.22 (0.87, 1.70)
Triglycerides, mmol/L						
<1.7	ref	ref	Ref	ref	ref	ref
≥1.7	1.29 (1.16, 1.44)	1.11 (0.99, 1.25)	1.33 (1.19, 1.49)	1.14 (1.00, 1.30)	1.02 (0.76, 1.38)	0.93 (0.65, 1.32)
High-density lipoprotein, mmol/L						
male≥1.03/female≥1.3	ref	ref	Ref	ref	ref	ref
male <1.03/female <1.3	1.17 (1.03, 1.34)	1.08 (0.93, 1.25)	1.23 (1.07, 1.42)	1.11 (0.94, 1.30)	0.76 (0.51, 1.11)	0.86 (0.54, 1.39)
eGFR, ml/min/1.73m^2^						
≥90	ref	ref	ref	ref	ref	ref
<90	1.35 (1.19, 1.54)	1.19 (1.03, 1.36)	1.36 (1.19, 1.57)	1.21 (1.04, 1.41)	1.39 (0.96, 2.00)	1.13 (0.75, 1.70)

*tHcy, total homocysteine; SBP, systolic blood pressure; eGFR, estimated glomerular filtration rate*.

*Adjusted for homocysteine, systolic blood pressure, body mass index, smoking status, diabetes, estimated glomerular filtration rate, triglyceride, high-density lipoprotein, physical intensity of job, anti-hypertensive drug, coronary heart disease*.

The associations between SBP, tHcy, and stroke risks are plotted in Figures A1, A2 in Supplementary Material. Further analyses confirmed the dose-relationship between SBP, tHcy levels, and the risk of the first stroke, ischemic and hemorrhagic strokes, either in the general population, or in age, sex, or center subgroups after adjusting for pertinent covariates.

### Stratified Analyses by Important Covariates

In the stratified analyses, diabetes (*P*-interaction = 0.02), HDL (*P*-interaction = 0.022), and LDL (*P*-interaction = 0.039) modified the association between tHcy and first stroke. Nevertheless, age, sex, BMI, smoking status, SBP, TG, physical intensity of the job, and history of CHD did not significantly modify the association between tHcy and first stroke or ischemic and hemorrhagic strokes (Table A4 in Supplementary Material).

### Additive Effects of tHcy and SBP on the First Stroke

The tHcy concentrations (per 5 μmol/L increment) were positively correlated with the risk of first stroke [1.06 (1.02, 1.10)], ischemic stroke [1.06 (1.01, 1.10)], but not hemorrhagic stroke [1.09 (0.98, 1.22)]. SBP levels (per 10 mmHg increment) were positively correlated with the risk of first stroke [1.13 (1.10, 1.16)], ischemic stroke [1.11 (1.08, 1.15)] and hemorrhagic stroke [1.26 (1.15, 1.37)]. However, homocysteine and SBP did not interact for the first stroke, ischemic or hemorrhagic strokes (Table 3).

**Table 3:** Independent and combined effects of systolic blood pressure and homocysteine on first stroke, ischemic and hemorrhagic stroke.

**SBP, mmHg/tHcy, μmol/L**	***N***	**Events (%)**	**Crude**	**Adjusted**	
			**OR (95% CI)**	**OR (95% CI)**	***N***
**First stroke**					
SBP, per 10 increment*	7,066	3,533 (50)	1.16 (1.13, 1.19)	1.13 (1.1, 1.16)	<0.001
tHcy, per 5 increment^†^	7,066	3,533 (50)	1.08 (1.04, 1.12)	1.06 (1.02, 1.10)	0.003
*P* for interaction			0.880	0.889	
*Joint effcts*					
SBP <140					
tHcy <10	498	194 (39)	ref	ref	
10 ≤ tHcy <15	1,040	432 (41.5)	1.23 (0.98, 1.55)	1.20 (0.95, 1.52)	0.134
tHcy ≥15	505	247 (48.9)	1.77 (1.35,2.34)	1.56 (1.17,2.08)	0.002
140 ≤ SBP <160					
tHcy <10	752	357 (47.5)	1.59 (1.25,2.04)	1.47 (1.14, 1.90)	0.003
10 ≤ tHcy <15	1,545	746 (48.3)	1.77 (1.41,2.22)	1.61 (1.27,2.04)	<0.001
tHcy ≥15	683	379 (55.5)	2.54 (1.95,3.30)	2.10 (1.58,2.77)	<0.001
SBP ≥160					
tHcy <10	423	240 (56.7)	2.48 (1.87,3.29)	2.19 (1.64,2.93)	<0.001
10 ≤ tHcy <15	1,018	574 (56.4)	2.60 (2.04,3.32)	2.13 (1.65,2.74)	<0.001
tHcy ≥15	602	364 (60.5)	3.27 (2.49,4.30)	2.64 (1.98,3.53)	<0.001
**Ischemic stroke**					
SBP, per 10 increment*	6,140	3,070 (50)	1.14 (1.11, 1.17)	1.11 (1.08, 1.15)	<0.001
tHcy, per 5 increment^†^	6,140	3,070 (50)	1.07 (1.03, 1.11)	1.06 (1.01, 1.10)	0.010
*P* for interaction			0.925	0.821	
*Joint effects*					
SBP <140					
tHcy <10	442	176 (39.8)	ref	ref	
10 ≤ tHcy <15	916	387 (42.2)	1.22 (0.96, 1.56)	1.22 (0.95, 1.57)	0.121
tHcy ≥15	459	230 (50.1)	1.79 (1.34,2.38)	1.60 (1.18,2.16)	0.002
140 ≤ SBP <160					
tHcy <10	641	315 (49.1)	1.64 (1.26,2.13)	1.52 (1.16,2.00)	0.002
10 ≤ tHcy <15	1,357	651 (48)	1.66 (1.30,2.11)	1.53 (1.19, 1.97)	<0.001
tHcy ≥15	598	325 (54.3)	2.29 (1.73,3.03)	1.93 (1.43,2.60)	<0.001
SBP ≥160					
tHcy <10	343	189 (55.1)	2.25 (1.66,3.06)	1.99 (1.45,2.73)	<0.001
10 ≤ tHcy <15	884	496 (56.1)	2.43 (1.88,3.15)	2.01 (1.53,2.63)	<0.001
tHcy ≥15	500	301 (60.2)	3.08 (2.29,4.13)	2.56 (1.88,3.51)	<0.001
**Hemorrhagic stroke**					
SBP, per 10 increment*	852	426 (50)	1.29 (1.19, 1.40)	1.26 (1.15, 1.37)	<0.001
tHcy, per 5 increment^†^	852	426 (50)	1.16 (1.03, 1.31)	1.09 (0.98, 1.22)	0.113
*P* for interaction			0.911	0.938	
*Joint effects*					
SBP <140					
tHcy <10	52	17 (32.7)	ref	ref	
10 ≤ tHcy <15	115	40 (34.8)	1.11 (0.51, 2.42)	0.96 (0.43,2.15)	0.930
tHcy ≥15	45	17 (37.8)	1.43 (0.55, 3.75)	1.10 (0.41,2.99)	0.849
140 ≤ SBP <160					
tHcy <10	100	38 (38)	1.30 (0.59, 2.88)	1.16 (0.52,2.60)	0.719
10 ≤ tHcy <15	173	88 (50.9)	2.81 (1.34, 5.86)	2.36 (1.10,5.04)	0.027
tHcy ≥15	83	53 (63.9)	5.52 (2.36, 12.92)	3.99 (1.61,9.92)	0.003
SBP ≥160					
tHcy <10	74	47 (63.5)	4.33 (1.91, 9.82)	3.68 (1.59,8.52)	0.002
10 ≤ tHcy <15	122	70 (57.4)	4.05 (1.83, 8.99)	3.31 (1.44,7.59)	0.005
tHcy ≥15	88	56 (63.6)	5.11 (2.23, 11.69)	3.77 (1.56,9.12)	0.003

*SBP, systolic blood pressure; tHcy, total homocysteine*.

*Conditioned on the matching factors of age, sex, and study site, and adjusted for body mass index, smoking status, diabetes, estimated glomerular filtration rate, triglyceride, high-density lipoprotein, physical intensity of job, antihypertensive drugs, coronary heart disease*.

* *Adjusted models included tHcy*.

† *Adjusted models included SBP*.

**Figure 2:**
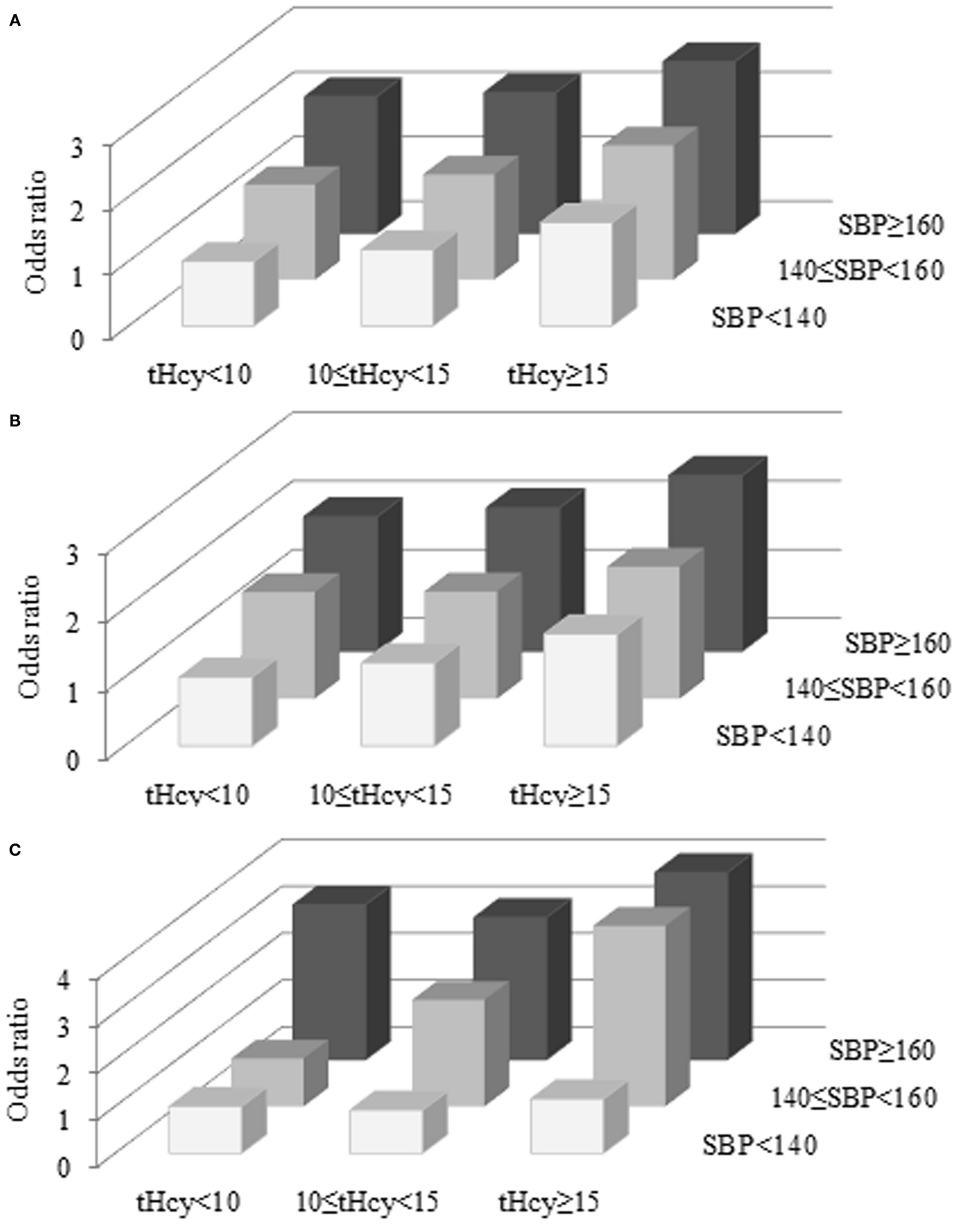
Combined effects of SBP and tHcy on the risk of first stroke **(A)**, ischemic stroke **(B)**, and hemorrhagic stroke **(C)**. tHcy, μmol/L; SBP, mmHg.

Table 3 and Figure 2 show that first stroke risk increased with both tHcy and SBP levels. The lowest first stroke risk was among subjects with the coexistence of tHcy < 10 μmol/L and SBP < 140 mmHg, while the highest risk of the first stroke was found among subjects with the coexistence of tHcy ≥ 15 μmol/L and SBP ≥ 160 mmHg after adjustments for potential covariates. Similar trends were found in ischemic and hemorrhagic strokes. SBP and tHcy had additive effects on the risk of first stroke and ischemic and hemorrhagic strokes.

### PARs of tHcy and Traditional Risk Factors for the First Stroke

**Figure 3:**
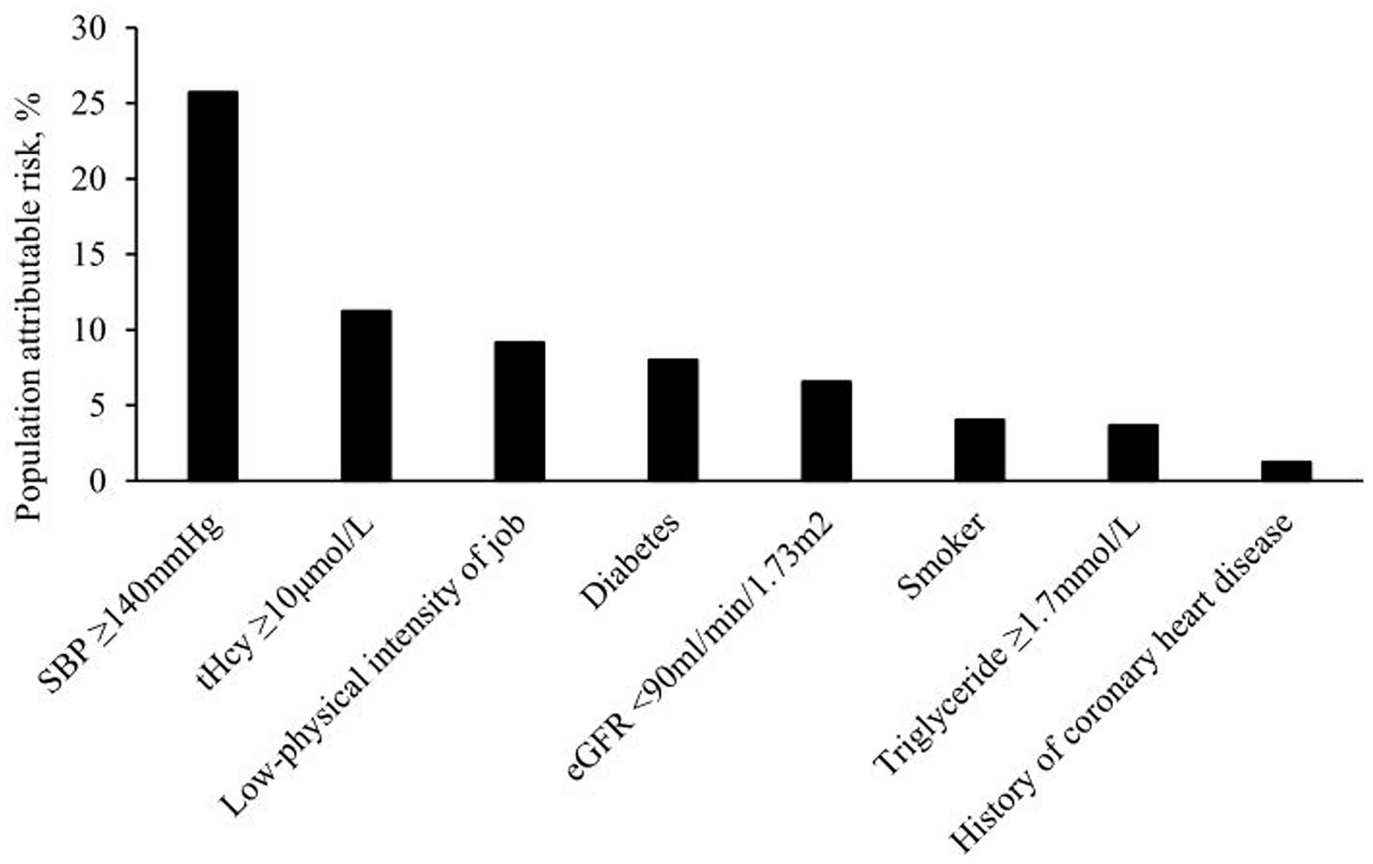
Population-attributable risks of traditional risk factors and tHcy for first stroke.

When we studied the first stroke, SBP was the most important risk factor (PAR, 25.73%), followed by tHcy (11.24%). In addition, a low-physical intensity job (9.17%), diabetes (8.01%), eGFR (6.57%), smoker (4.04%), TG (3.68%), and CHD (1.88%) contributed significantly to the stroke burden. The combined PAR of SBP and tHcy for the first stroke was 34.08%. The total proportion of the first stroke that could be attributed to a combination of these 11 examined risk factors was 54.88% (Table 4, Figure 3).

**Table 4:** Adjusted odds ratio and population attributable risks for first stroke, ischemic stroke, and hemorrhagic stroke.

	**First stroke**		**Ischemic stroke**		**Hemorrhagic stroke**	
	**OR (95% CI)**	**PAR% (95% CI)**	**OR (95% CI)**	**PAR% (95% CI)**	**OR (95% CI)**	**PAR% (95% CI)**
Individual risk factors						
tHcy ≥10 μmol/L	1.17 (1.03, 1.33)	11.24% (1.58, 18.93)	1.14 (1.00, 1.31)	9.8% (-0.62, 18.46)	1.35 (0.91,2.01)	19.92% (-8.31,40.86)
SBP ≥140 mmHg	1.52 (1.34, 1.71)	25.73% (19.18,32.54)	1.43 (1.25, 1.62)	22.21% (14.23,28.53)	2.63 (1.75,3.94)	51.15% (36.45,65.21)
Body mass index ≥28 kg/m^2^	1.01 (0.90, 1.14)	0.33% (−3.22, 3.45)	1.02 (0.90, 1.16)	0.66% (−2.76, 3.61)	0.99 (0.71, 1.37)	−0.46% (−12.57, 10.2)
Smoker	1.16 (1.01, 1.34)	4.04% (0.11, 7.38)	1.17 (1.00, 1.36)	4.08% (0.13,7.8)	1.03 (0.69, 1.53)	0.87% (−13.7, 12.06)
Diabetes	1.47 (1.30, 1.65)	8.01% (5.46, 10.67)	1.58 (1.39, 1.80)	9.63% (7.03, 12.34)	1.02 (0.70, 1.48)	0.35% (−7.22, 7.24)
Triglyceride ≥1.7 mmol/L	1.13 (1.01, 1.27)	3.68% (0.29, 7.03)	1.16 (1.02, 1.32)	4.39% (0.71, 8.19)	0.93 (0.65, 1.32)	−2.31% (−13.06, 7.72)
High-density lipoprotein (male <1.03/female <1.3 mmo/L)	1.07 (0.92, 1.24)	1.05% (−1.5, 3.39)	1.10 (0.94, 1.30)	1.6% (−0.97, 3.97)	0.83 (0.52, 1.33)	−2.64% (−10.85, 3.53)
Low-density lipoprotein ≥3.4 mmol/L	1.08 (0.97, 1.20)	3.12% (−1.64, 7.35)	1.05 (0.93, 1.18)	1.94% (−2.7, 7.24)	1.29 (0.94, 1.79)	9.66% (−2.01, 20.9)
eGFR <90 ml/min/1.73 m^2^	1.269 (1.10, 1.44)	6.57% (2.49, 10.34)	1.28 (1.11, 1.48)	7.17% (2.89, 11.19)	1.22 (0.81, 1.82)	5.23% (−6.56, 15.44)
Low-physical intensity of job	1.16 (1.04, 1.30)	9.17% (2.73, 15.82)	1.18 (1.04, 1.33)	9.86% (2.73, 16.7)	1.05 (0.74, 1.48)	2.95% (−22.7, 23.33)
History of coronary heart disease	1.22 (1.02, 1.46)	1.88% (0.12, 3.41)	1.25 (1.04, 1.52)	2.1% (0.35, 3.99)	0.93 (0.56, 1.54)	−0.72% (−5.77, 4.06)
Combinations of risk factors						
SBP ≥140 mmHg and tHcy ≥10 μmol/L		34.08% (24.81, 41.98)		29.83% (17.62, 38.79)		60.91% (40.39, 76.54)
All risk factors above		54.88% (46.94, 61.56)		53.79% (45.53, 61.37)		65.24% (45.35, 79.16)
All risk factors but without tHcy		49.29% (42.27, 55.70)		48.87% (41.20, 55.16)		56.76% (34.98, 72.62)

*SBP, systolic blood pressure; tHcy, total homocysteine; eGFR, estimated glomerular filtration rate*.

*Adjusted for body mass index, smoking status, diabetes, estimated glomerular filtration rate, triglyceride, high-density lipoprotein, low-density lipoprotein, physical intensity of job, anti-hypertensive drug, coronary heart disease*.

When we restricted the analyses to ischemic stroke, similar contributions of individual and combined risk factors were found (Table 4). SBP explained 22.21% of ischemic stroke, followed by tHcy (PAR, 9.89%). SBP and tHcy combined to explain 29.83% of the ischemic strokes. The combination of all 11 involved risk factors explained 53.79% of ischemic strokes. Compared with ischemic stroke, hemorrhagic stroke was to a greater extent explained by SBP (PAR, 51.15%). SBP and tHcy combined to explain 60.91% of hemorrhagic stroke, and all involved eleven risk factors combined to explain 65.24% of hemorrhagic stroke (Table 4).

We summarized 11 studies on PARs of risk factors for the first stroke in Table A5 in Supplementary Material. Participants were recruited from different regions, most of them were hospital based, with different study designs and different risk factors. The major factors affecting the risk of the first stroke can be divided into conventional cardiovascular factors, family history, lifestyle, dietary habits, education, and other aspects. The reported PAR of hypertension for the first stroke in different studies varies widely.

## Discussion

To our knowledge, this is by far one of the largest prospective nested case-control studies of this kind to confirm that with increasing tHcy and SBP levels, the risks of the first stroke, ischemic and hemorrhagic strokes significantly increase. We found that more than half of the first strokes in this study population were attributable to established modifiable risk factors. Baseline SBP and tHcy were two of the most important risk factors and can independently and additively increase the risk of the first stroke. However, the contribution of modifiable risk factors differed between ischemic and hemorrhagic strokes.

Consistent with previous studies, (5, 8, 13, 22) our findings showed that SBP and tHcy were positively correlated with the first stroke. It is universally acknowledged that high SBP is the leading risk factor for all stroke types (1, 5, 23). Among different populations, however, data on the association of tHcy with the risk of stroke subtypes remain limited. Huo et al. (8) noted a strong association of HHcy with the risk of first stroke and the ischemic subtype, but the analysis was underpowered for assessing hemorrhagic stroke among the Chinese population. A nested case-control study of Japanese adults showed positive relationships between HHcy and the risks of first stroke and ischemic stroke (22). However, it did not investigate the interaction between tHcy and blood pressure. For hemorrhagic stroke in that study, despite the lack of statistical significance between hemorrhagic stroke and HHcy, a trend in higher quartiles of tHcy with an increased risk of hemorrhagic stroke was observed. We found a significant association of tHcy ≥ 15 μmol/L and SBP ≥ 140 mmHg with hemorrhagic stroke. Nevertheless, studies on the association between HHcy and hemorrhagic stroke are inconsistent (18, 24–28). Overall, studies suggest positive associations of HHcy with the risk of the first stroke.

^*****^Furthermore, we reported that HHcy and high SBP additively increased the risks of the first stroke, ischemic and hemorrhagic stroke. We speculate that the effect of HHcy and SBP on stroke may stem from their multifaceted biological pathways. Pathophysiologically, HHcy damages the vascular structure through different isoforms of oxidative stress, inflammation, and apoptosis promotes atherosclerotic properties and atherosclerotic plaque rupture, and subsequently increases stroke risk (29). In addition, the adverse effects of HHcy in stroke are caused by the upregulation of angiotensin II, subsequently leading to hypertension (30). Hypertension promotes stroke through activation of angiotensin II and angiotensin type 1 receptor in the blood vessels and increases vasoconstriction, causing vascular wall damage and blood–brain barrier disruption (31). In addition, HHcy upregulates pathogenic genes *via* DNA demethylation to increase vascular remodeling and hypertension (32). Interestingly, mild and moderate HHcy levels primarily affect the epigenetic regulation of gene expression through the interference of transmethylation reactions, while severe HHcy might be more destructive through oxidative stress, inflammation, and apoptosis (29, 33). The multiple pathogenesis involved in the association of tHcy with systolic hypertension may explain their additive effects for stroke.

Our study provides further evidence that SBP and HHcy are two of the most important risk factors for the first stroke in Chinese adults in the rural areas. Consistent with other studies (5, 13), high SBP was the strongest contributor for the first stroke and its aetiologic subtypes. HHcy was the second most important contributor for the first stroke in our study, while it was not recruited to the panel of risk factors in the previous studies. The prevalence of HHcy was high in China (7), and preventive control of HHcy is an effective approach to decrease the burden of stroke (8), therefore, we should pay additional attention to HHcy, the leading risk factor for the first stroke. The PARs of risk factors in our study were considerably lower than those reported previously. Several explanations are possible. First, the risk factors included in different studies are not completely consistent, and the criteria for defining risk factors are different. Second, there were differences regarding whether risk factors were assessed before or after the occurrence of stroke. Risk factors might be raised in the acute stroke phase, whereas before the stroke onset might be lower. Therefore, previous case-control studies may have overestimated the PARs. Third, we highlighted the difference in population characteristics. Previous studies were mostly hospital-based Western populations, while our study focused on the first stroke in the community-based Chinese population. Finally, the lack of data on the psychosocial factors and socioeconomic status probably did not materially influence our total PAR because causality for these factors has not been established.

The strengths of our study include its large real-world sample, economical nature, nested case-control design, and ability to adjust for the traditional risk factors for stroke. Several potential limitations should be addressed. First, our findings cannot establish causality. Moreover, the study population was Chinese adults aged 35 years and over in the rural areas, therefore, our findings cannot be extrapolated to other populations. Second, this study did not evaluate the associations of stroke with diet, hypertensive medication use, or supplementation with B vitamins in the follow-up period that may affect the outcome of stroke (34, 35). Third, we were unable to distinguish subtypes of ischemic or hemorrhagic stroke owing to the lack of imaging data. The associations of tHcy with arterial territories of stroke are worth further study.

In conclusion, consistent associations between tHcy and first stroke and ischemic and hemorrhagic stroke were observed after adjustment for potential covariates. This study emphasizes that SBP and tHcy are two of the most important risk factors and have independent and additive effects on the risk of the first stroke, ischemic and hemorrhagic stroke. The findings underscore the importance of screening and controlling high SBP and HHcy among the Chinese population in a rural area in order to further reduce the population burden of the first stroke.

## Data Availability Statement

The original contributions presented in the study are included in the article/Supplementary Material, further inquiries can be directed to the corresponding authors.

## Ethics Statement

The studies involving human participants were reviewed and approved by the Ethics Committee of the Institute of Biomedicine, Anhui Medical University, Hefei, China. The patients/participants provided their written informed consent to participate in this study.

## Author Contributions

XW, XX, and CZ gave the conception and methodology. FZ, CL, LY, YW, YSh, GZ, ZD, JC, and JK helped in the investigation. CL, LY, and JL were involved in data curation and analysis. YSo and LL helped in the validation. FZ, PZ, and CZ wrote the manuscript. XX and CZ helped in the supervision and with the financial support. All the authors read and approved the final manuscript.

## Funding

This study was supported by funding from the following: the National Key Research and Development Program (2016YFE0205400, 2018ZX09739010, and 2018ZX09301034003), the Science and Technology Planning Project of Guangzhou, China (201707020010), the Science, Technology, and Innovation Committee of Shenzhen (GJHS20170314114526143 and JSGG20180703155802047), the Economic, Trade and Information Commission of Shenzhen Municipality (20170505161556110, 20170505160926390, and 201705051617070), and the Special Fund of Foshan Summit Plan (2019D044 and 2020A022).

## Conflict of Interest

The authors declare that the research was conducted in the absence of any commercial or financial relationships that could be construed as a potential conflict of interest.

## Publisher's Note

All claims expressed in this article are solely those of the authors and do not necessarily represent those of their affiliated organizations, or those of the publisher, the editors and the reviewers. Any product that may be evaluated in this article, or claim that may be made by its manufacturer, is not guaranteed or endorsed by the publisher.
