# Supplementary material for: The Relative Contribution of Plasma Homocysteine Levels vs. Traditional Risk Factors to the First Stroke: A Nested Case-Control Study in Rural China
Source: Front Med (Lausanne). 2022 Jan 20;8:727418. doi: 10.3389/fmed.2021.727418 (PMC8811122; doi:10.3389/fmed.2021.727418)
Supplement: Supplementary file 1 [file Data_Sheet_1.docx]

**
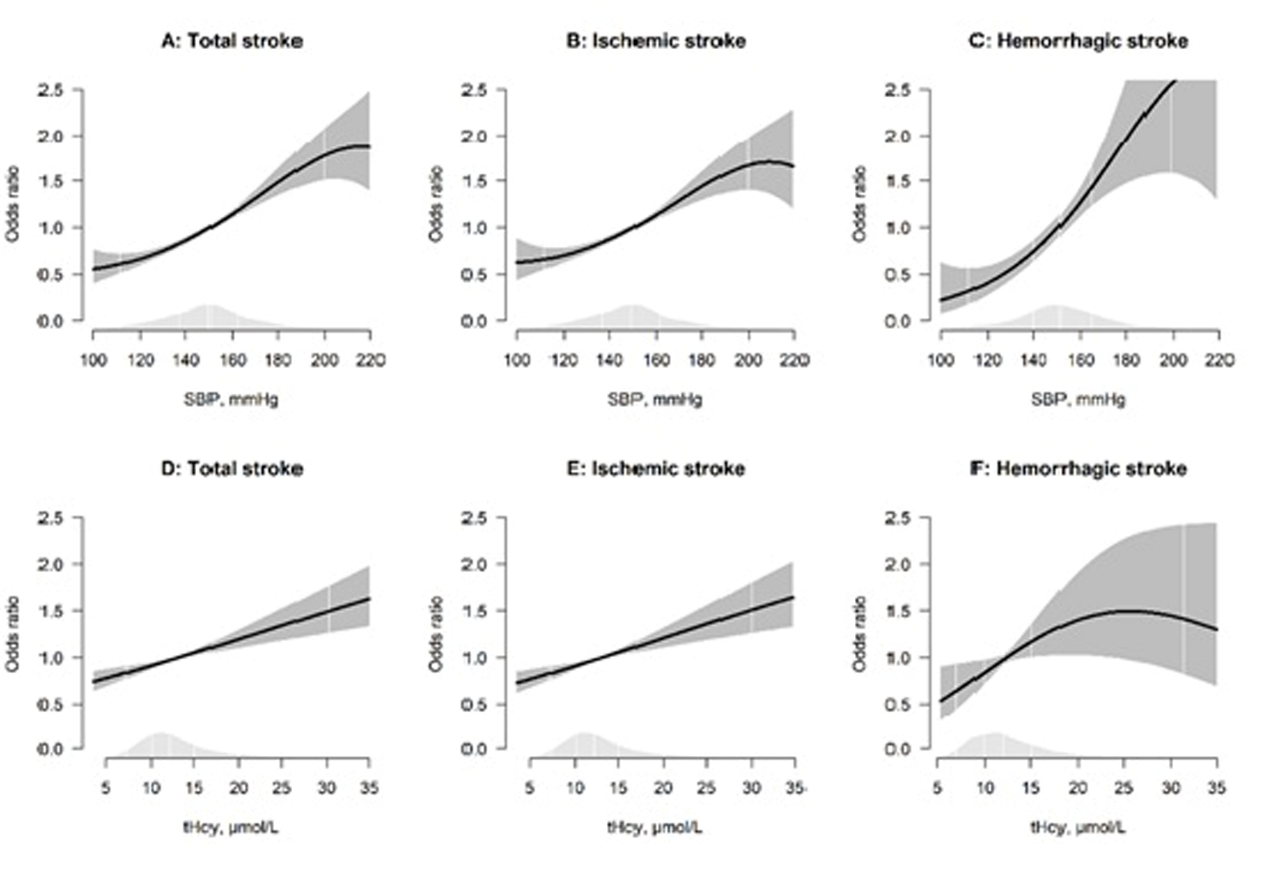
 Appendix Figure 1**. **The associations between first stroke and baseline SBP or tHcy for the general population.** In addition to the matching factors of age, sex, and study site, the splines (A, B, C) also adjusted for tHcy, BMI, smoking, diabetes, eGFR, triglyceride, HDL, physical intensity of job, antihypertensive drugs, CHD; the splines (D, E, F) also adjusted for SBP, BMI, smoking, diabetes, eGFR, triglyceride, HDL, physical intensity of job, antihypertensive drugs, CHD.

**
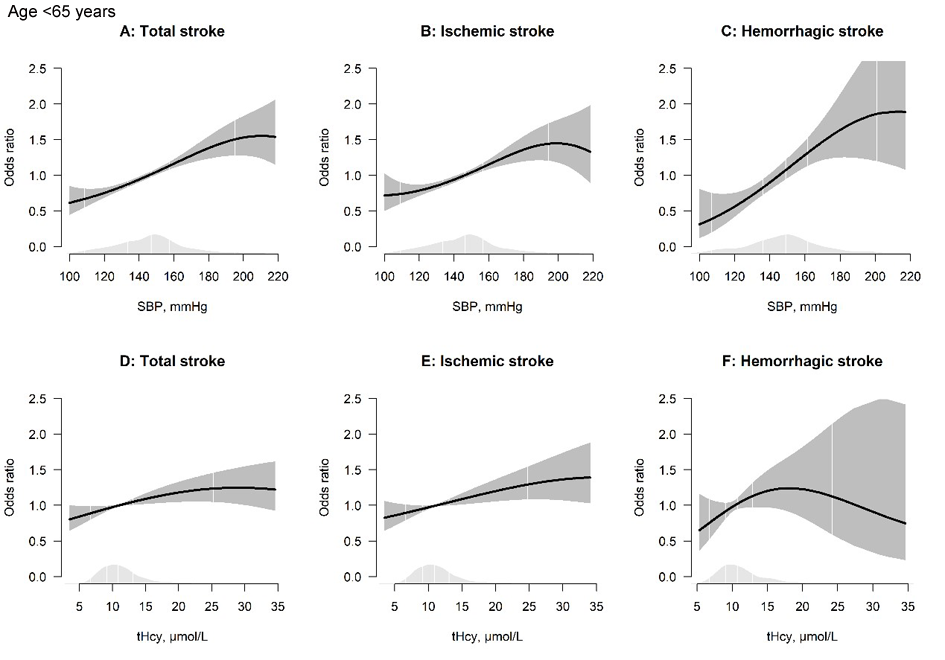

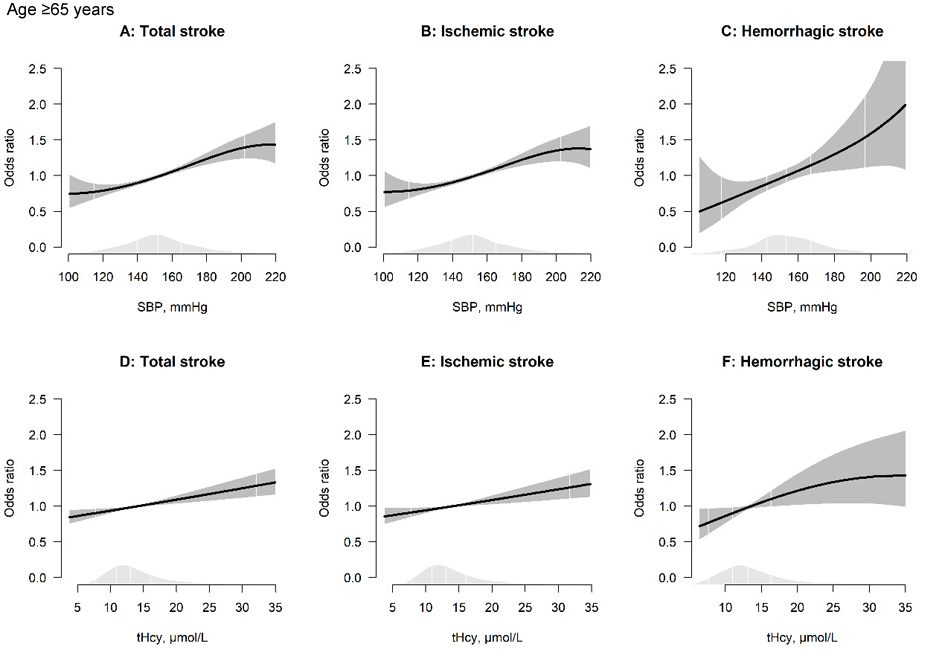

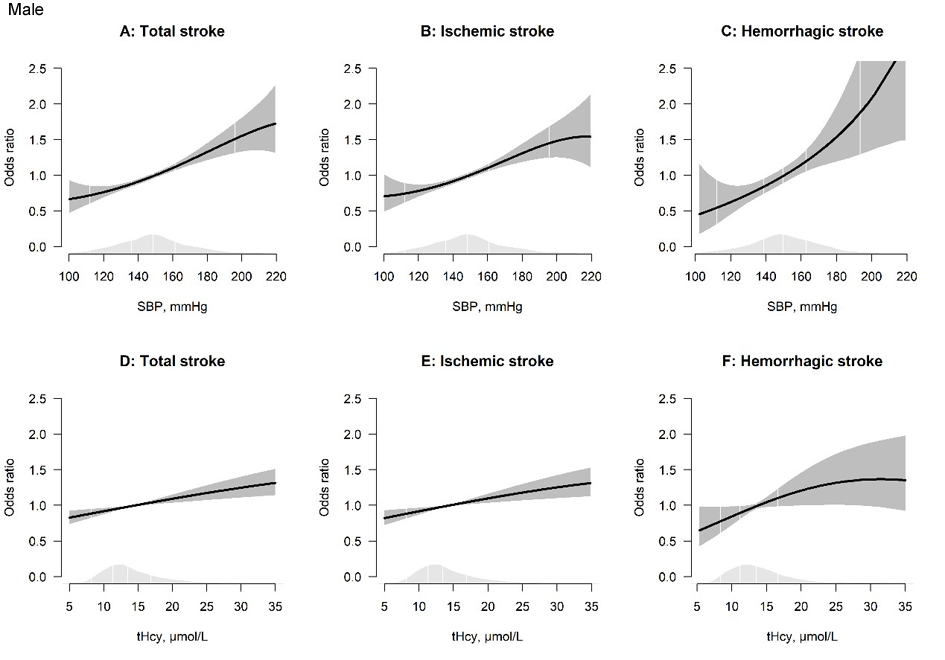

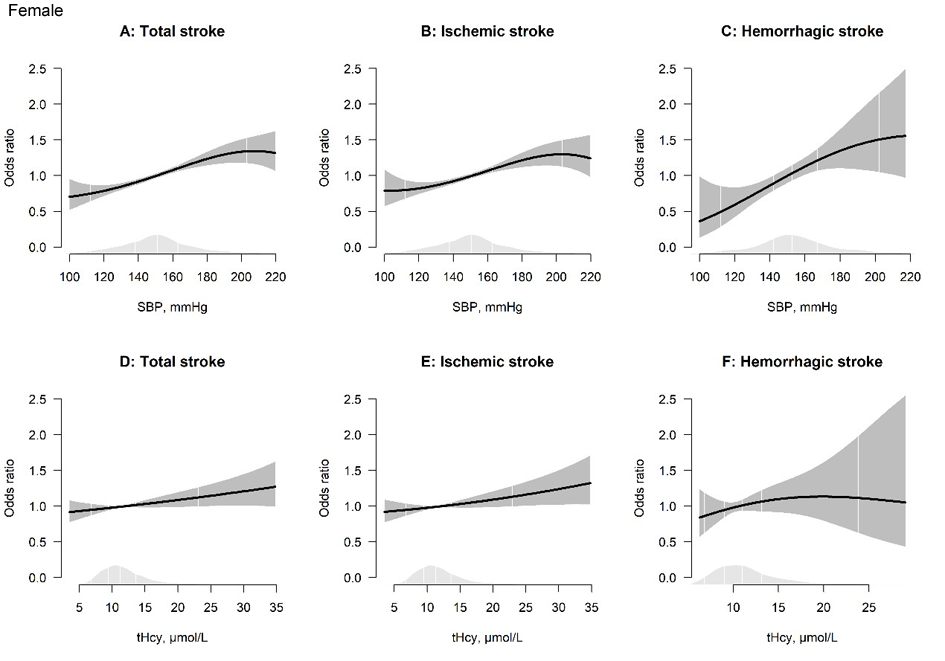
**

**
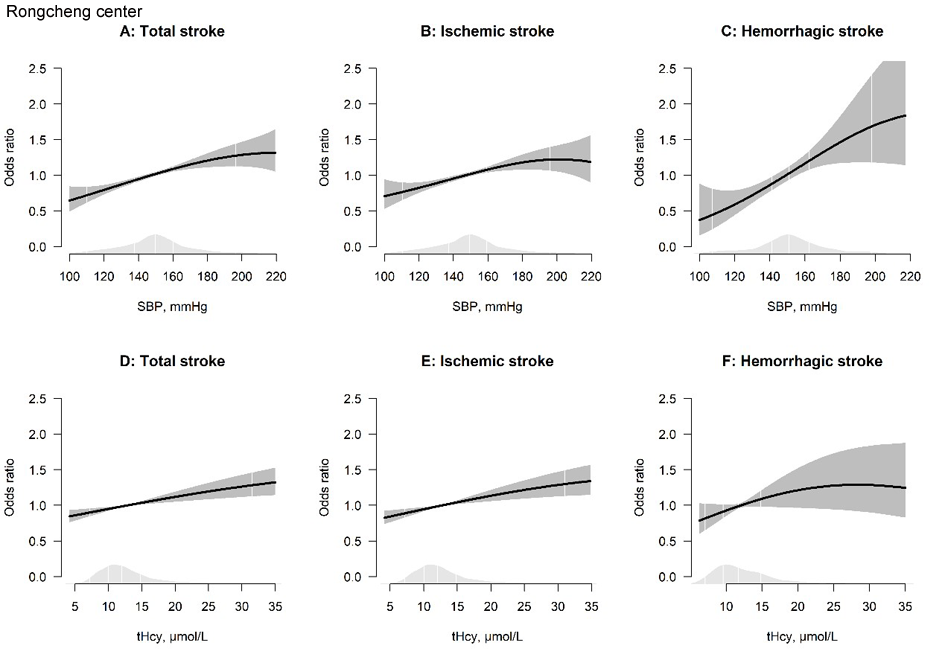

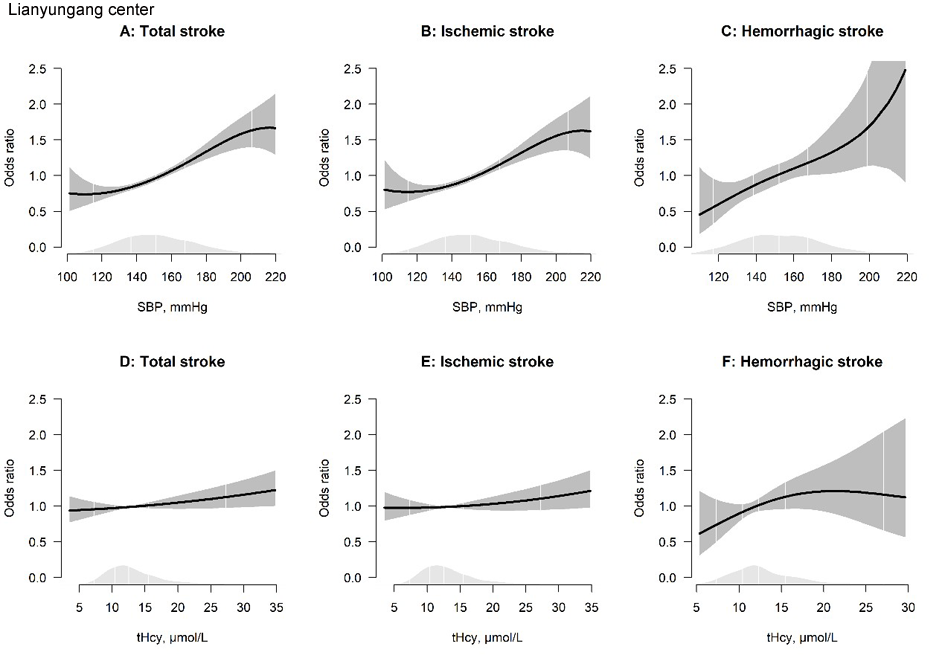
**

**Appendix Figure 2. The associations between first stroke and baseline SBP or tHcy for the age (<65, ≥65 years), sex (male, female), center (Rongcheng, Lianyungang) subgroups, respectively.** In addition to the matching factors of age, sex, and study site, the splines (A, B, C) also adjusted for tHcy, BMI, smoking, diabetes, eGFR, triglyceride, HDL, physical intensity of job, antihypertensive drugs, CHD; the splines (D, E, F) also adjusted for SBP, BMI, smoking, diabetes, eGFR, triglyceride, HDL, physical intensity of job, antihypertensive drugs, CHD.

**Appendix Table 1.** The associations of tHcy and traditional risk factors with the risk of first stroke, ischemic and hemorrhagic stroke by sex subgroups.

|  | First stroke | | Ischemic stroke | | Hemorrhagic stroke | |
| --- | --- | --- | --- | --- | --- | --- |
|  | Crude | Adjusted | Crude | Adjusted | Crude | Adjusted |
| Female |  |  |  |  |  |  |
| tHcy, μmol/L |  |  |  |  |  |  |
| <10 | ref | ref | ref | ref | ref | ref |
| 10-<15 | 1.19 (1.02,1.38) | 1.19 (1.02,1.40) | 1.19 (1.01,1.4) | 1.20 (1.01,1.42) | 1.14 (0.73,1.8) | 1.29 (0.77,2.16) |
| ≥15 | 1.43 (1.16,1.77) | 1.38 (1.10,1.73) | 1.44 (1.15,1.8) | 1.39 (1.09,1.77) | 1.49 (0.76,2.9) | 1.29 (0.60,2.79) |
| SBP, mmHg |  |  |  |  |  |  |
| <140 | ref | ref | ref | ref | ref | ref |
| 140-<160 | 1.34 (1.13,1.59) | 1.27 (1.07,1.52) | 1.27 (1.06,1.51) | 1.20 (0.99,1.44) | 2.45 (1.36,4.40) | 2.40 (1.29,4.48) |
| ≥160 | 1.97 (1.62,2.38) | 1.70 (1.39,2.08) | 1.84 (1.50,2.26) | 1.57 (1.27,1.96) | 3.57 (1.92,6.62) | 3.53 (1.79,6.94) |
| BMI, kg/m^2^ |  |  |  |  |  |  |
| <28 | ref | ref | ref | ref | ref | ref |
| ≥28 | 1.14 (0.99,1.31) | 1.01 (0.87,1.17) | 1.22 (1.05,1.41) | 1.07 (0.92,1.26) | 0.86 (0.59,1.26) | 0.77 (0.49,1.21) |
| Smoking status |  |  |  |  |  |  |
| Never | ref | ref | ref | ref | ref | ref |
| Ever | 1.29 (0.86,1.92) | 1.39 (0.91,2.11) | 1.15 (0.75,1.77) | 1.3 (0.83,2.04) | 3.5 (0.73,16.85) | 2.93 (0.55,15.6) |
| Diabetes |  |  |  |  |  |  |
| No | ref | ref | ref | ref | ref | ref |
| Yes | 1.56 (1.34,1.81) | 1.45 (1.24,1.7) | 1.72 (1.46,2.02) | 1.6 (1.35,1.89) | 0.86 (0.55,1.34) | 0.91 (0.54,1.51) |
| TC, mmol/L |  |  |  |  |  |  |
| <5.2 | ref | ref | ref | ref | ref | ref |
| ≥5.2 | 0.96 (0.84,1.1) | 0.95 (0.81,1.1) | 0.94 (0.81,1.09) | 0.94 (0.8,1.11) | 1.16 (0.77,1.73) | 0.92 (0.56,1.5) |
| TG, mmol/L |  |  |  |  | ref | ref |
| <1.7 | ref | ref | ref | ref | 0.84 (0.57,1.24) | 0.91 (0.57,1.46) |
| ≥1.7 | 1.22 (1.07,1.39) | 1.06 (0.92,1.24) | 1.28 (1.11,1.47) | 1.08 (0.91,1.27) |  |  |
| HDL, mmol/L | ref | ref | ref | ref | ref | ref |
| male≥1.03/female≥1.3 | 1.13 (0.97,1.31) | 1.05 (0.88,1.25) | 1.22 (1.03,1.43) | 1.12 (0.93,1.34) | 0.52 (0.32,0.87) | 0.59 (0.32,1.08) |
| male<1.03/female<1.3 | 1.19 (1.02,1.38) | 1.19 (1.02,1.40) | 1.19 (1.01,1.4) | 1.20 (1.01,1.42) | 1.14 (0.73,1.8) | 1.29 (0.77,2.16) |
| Male |  |  |  |  |  |  |
| tHcy, μmol/L |  |  |  |  |  |  |
| <10 | ref | ref | ref | ref | ref | ref |
| 10-15 | 0.97 (0.77,1.22) | 0.94 (0.74,1.19) | 0.88 (0.68,1.12) | 0.85 (0.66,1.11) | 1.77 (0.90,3.49) | 1.79 (0.84,3.80) |
| ≥15 | 1.49 (1.16,1.90) | 1.36 (1.04,1.76) | 1.32 (1.01,1.72) | 1.24 (0.94,1.65) | 3.15 (1.52,6.53) | 2.51 (1.09,5.76) |
| SBP, mmHg |  |  |  |  |  |  |
| <140 | ref | ref | ref | ref | ref | ref |
| 140-160 | 1.57 (1.31,1.88) | 1.55 (1.28,1.87) | 1.51 (1.25,1.83) | 1.49 (1.22,1.82) | 2.28 (1.27,4.10) | 2.02 (1.07,3.81) |
| ≥160 | 2.25 (1.83,2.77) | 2.06 (1.66,2.56) | 2.07 (1.66,2.58) | 1.91 (1.51,2.41) | 4.06 (2.15,7.64) | 3.41 (1.72,6.73) |
| BMI, kg/m^2^ |  |  |  |  |  |  |
| <28 | ref | ref | ref | ref | ref | ref |
| ≥28 | 1.24 (1.04,1.49) | 0.99 (0.81,1.2) | 1.15 (0.94,1.4) | 0.93 (0.75,1.15) | 2.10 (1.25,3.52) | 1.43 (0.79,2.58) |
| Smoking status |  |  |  |  |  |  |
| Never | ref | ref | ref | ref | ref | ref |
| Ever | 1.10 (0.95,1.26) | 1.13 (0.97,1.32) | 1.11 (0.95,1.29) | 1.14 (0.97,1.35) | 1.08 (0.74,1.57) | 0.96 (0.62,1.5) |
| Diabetes |  |  |  |  |  |  |
| No | ref | ref | ref | ref | ref | ref |
| Yes | 1.59 (1.33,1.91) | 1.44 (1.19,1.74) | 1.68 (1.38,2.04) | 1.51 (1.23,1.85) | 1.21 (0.7,2.08) | 1.11 (0.6,2.08) |
| TC, mmol/L |  |  |  |  |  |  |
| <5.2 | ref | ref | ref | ref | ref | ref |
| ≥5.2 | 1.06 (0.91,1.22) | 1.02 (0.87,1.2) | 1.01 (0.86,1.18) | 0.96 (0.81,1.15) | 1.38 (0.9,2.1) | 1.59 (0.97,2.61) |
| TG, mmol/L |  |  |  |  |  |  |
| <1.7 | ref | ref | ref | ref | ref | ref |
| ≥1.7 | 1.43 (1.20,1.70) | 1.22 (1.01,1.48) | 1.43 (1.19,1.73) | 1.28 (1.04,1.58) | 1.39 (0.86,2.26) | 0.87 (0.47,1.61) |
| HDL, mmol/L |  |  |  |  |  |  |
| male≥1.03/female≥1.3 | ref | ref | ref | ref | ref | ref |
| male<1.03/female<1.3 | 1.35 (1.02,1.79) | 1.18 (0.86,1.61) | 1.29 (0.95,1.76) | 1.1 0(0.79,1.54) | 1.73 (0.82,3.63) | 1.98 (0.82,4.83) |

Adjusted for tHcy, SBP, BMI, smoking, diabetes, eGFR, triglyceride, HDL, physical intensity of job, antihypertensive drugs, CHD.

**Appendix Table 2.** The associations of tHcy and traditional risk factors with first stroke, ischemic and hemorrhagic stroke by age subgroups.

|  | First stroke | | Ischemic stroke | | Hemorrhagic stroke | |
| --- | --- | --- | --- | --- | --- | --- |
|  | Crude | Adjusted | Crude | Adjusted | Crude | Adjusted |
| Age ≥ 65 years |  |  |  |  |  |  |
| tHcy, μmol/L |  |  |  |  |  |  |
| <10 | ref | ref | ref | ref | ref | ref |
| 10-<15 | 1.02 (0.85,1.22) | 1.00 (0.83,1.21) | 0.99 (0.82,1.2) | 0.97 (0.79,1.19) | 1.37 (0.79,2.37) | 1.42 (0.79,2.54) |
| ≥15 | 1.45 (1.18,1.78) | 1.33 (1.07,1.66) | 1.37 (1.10,1.7) | 1.25 (0.99,1.58) | 2.50 (1.33,4.69) | 2.30 (1.15,4.61) |
| SBP, mmHg |  |  |  |  |  |  |
| <140 | ref | ref | ref | ref | ref | ref |
| 140-<160 | 1.33 (1.14,1.56) | 1.30 (1.10,1.53) | 1.29 (1.09,1.53) | 1.25 (1.05,1.49) | 1.80 (1.03,3.16) | 1.78 (0.99,3.2) |
| ≥160 | 1.92 (1.61,2.28) | 1.74 (1.45,2.08) | 1.82 (1.52,2.19) | 1.65 (1.36,2.01) | 2.89 (1.62,5.14) | 2.57 (1.40,4.7) |
| BMI, kg/m^2^ |  |  |  |  |  |  |
| <28 | ref | ref | ref | ref | ref | ref |
| ≥28 | 1.22 (1.06,1.41) | 1.05 (0.9,1.22) | 1.25 (1.08,1.46) | 1.07 (0.91,1.27) | 1.07 (0.71,1.59) | 0.87 (0.56,1.37) |
| Smoking status |  |  |  |  |  |  |
| Never | ref | ref | ref | ref | ref | ref |
| Ever | 1.08 (0.92,1.27) | 1.12 (0.95,1.32) | 1.07 (0.9,1.28) | 1.12 (0.93,1.34) | 1.24 (0.79,1.96) | 1.08 (0.65,1.8) |
| Diabetes |  |  |  |  |  |  |
| No | ref | ref | ref | ref | ref | ref |
| Yes | 1.58 (1.36,1.83) | 1.47 (1.26,1.71) | 1.66 (1.42,1.94) | 1.54 (1.31,1.82) | 1.09 (0.68,1.73) | 1.07 (0.65,1.78) |
| TC, mmol/L |  |  |  |  |  |  |
| <5.2 | ref | ref | ref | ref | ref | ref |
| ≥5.2 | 1.07 (0.94,1.21) | 1.07 (0.93,1.22) | 1.06 (0.92,1.21) | 1.06 (0.91,1.23) | 1.17 (0.79,1.72) | 1.15 (0.74,1.79) |
| TG, mmol/L |  |  |  |  |  |  |
| <1.7 | ref | ref | ref | ref | ref | ref |
| ≥1.7 | 1.36 (1.18,1.57) | 1.17 (1.01,1.37) | 1.4 (1.2,1.62) | 1.19 (1.01,1.41) | 1.02 (0.67,1.56) | 1.01 (0.62,1.63) |
| HDL, mmol/L |  |  |  |  |  |  |
| male≥1.03/female≥1.3 | ref | ref | ref | ref | ref | ref |
| male<1.03/female<1.3 | 1.28 (1.07,1.52) | 1.13 (0.93,1.38) | 1.34 (1.11,1.61) | 1.17 (0.95,1.44) | 0.78 (0.44,1.38) | 0.85 (0.44,1.64) |
| Age < 65 years |  |  |  |  |  |  |
| tHcy, μmol/L |  |  |  |  |  |  |
| <10 | ref | ref | ref | ref | ref | ref |
| 10-15 | 1.20 (1.01,1.44) | 1.20 (1.00,1.45) | 1.17 (0.97,1.41) | 1.20 (0.98,1.47) | 1.28 (0.76,2.14) | 1.37 (0.74,2.51) |
| ≥15 | 1.54 (1.18,2.00) | 1.51 (1.14,2.01) | 1.54 (1.16,2.04) | 1.58 (1.16,2.15) | 1.58 (0.78,3.21) | 1.26 (0.52,3.06) |
| SBP, mmHg |  |  |  |  |  |  |
| <140 | ref | ref | ref | ref | ref | ref |
| 140-160 | 1.60 (1.32,1.94) | 1.47 (1.20,1.80) | 1.5 (1.22,1.84) | 1.36 (1.09,1.7) | 3.14 (1.69, 5.85) | 2.78 (1.41, 5.47) |
| ≥160 | 2.45 (1.92,3.12) | 2.05 (1.58,2.65) | 2.2 (1.69,2.87) | 1.81 (1.36,2.4) | 5.22 (2.58,10.57) | 4.99 (2.26,11.05) |
| BMI, kg/m^2^ |  |  |  |  |  |  |
| <28 | ref | ref | ref | ref | ref | ref |
| ≥28 | 1.12 (0.94,1.32) | 0.94 (0.78,1.13) | 1.1 (0.92,1.33) | 0.95 (0.77,1.16) | 1.37 (0.87,2.17) | 1.07 (0.6,1.88) |
| Smoking status |  |  |  |  |  |  |
| Never | ref | ref | ref | ref | ref | ref |
| Ever | 1.2 (0.94,1.52) | 1.31 (1.01,1.69) | 1.22 (0.94,1.58) | 1.33 (1,1.76) | 1.05 (0.58,1.9) | 0.95 (0.46,1.97) |
| Diabetes |  |  |  |  |  |  |
| No | ref | ref | ref | ref | ref | ref |
| Yes | 1.56 (1.29,1.88) | 1.4 (1.14,1.72) | 1.78 (1.44,2.2) | 1.59 (1.27,1.99) | 0.88 (0.53,1.45) | 0.75 (0.4,1.41) |
| TC, mmol/L |  |  |  |  |  |  |
| <5.2 | ref | ref | ref | ref | ref | ref |
| ≥5.2 | 0.92 (0.78,1.08) | 0.88 (0.73,1.06) | 0.85 (0.71,1.01) | 0.82 (0.67,1) | 1.39 (0.89,2.18) | 1.42 (0.81,2.5) |
| TG, mmol/L |  |  |  |  |  |  |
| <1.7 | ref | ref | ref | ref | ref | ref |
| ≥1.7 | 1.23 (1.05,1.44) | 1.04 (0.86,1.25) | 1.27 (1.07,1.52) | 1.08 (0.88,1.32) | 1.05 (0.68,1.61) | 0.79 (0.44,1.4) |
| HDL, mmol/L |  |  |  |  |  |  |
| male≥1.03/female≥1.3 | ref | ref | ref | ref | ref | ref |
| male<1.03/female<1.3 | 1.05 (0.85,1.29) | 1.00 (0.78,1.27) | 1.10 (0.88,1.38) | 1.01 (0.78,1.32) | 0.78 (0.44,1.38) | 0.96 (0.45,2.03) |

Adjusted for tHcy, SBP, BMI, smoking, diabetes, eGFR, triglyceride, HDL, physical intensity of job, antihypertensive drugs, CHD.

**Appendix Table 3.** The associations of tHcy and traditional risk factors with first stroke, ischemic and hemorrhagic stroke by center subgroups.

|  | First stroke | | Ischemic stroke | | Hemorrhagic stroke | |
| --- | --- | --- | --- | --- | --- | --- |
|  | Crude | Adjusted | Crude | Adjusted | Crude | Adjusted |
| Center: Rongcheng |  |  |  |  |  |  |
| tHcy, μmol/L |  |  |  |  |  |  |
| <10 | ref | ref | ref | ref | ref | ref |
| 10-<15 | 1.07 (0.85,1.33) | 1.09 (0.86,1.38) | 1.03 (0.81,1.31) | 1.04 (0.81,1.35) | 1.32 (0.71,2.43) | 1.48 (0.75,2.92) |
| ≥15 | 1.45 (1.12,1.88) | 1.37 (1.03,1.82) | 1.38 (1.04,1.82) | 1.29 (0.95,1.75) | 2.10 (0.99,4.46) | 2.08 (0.91,4.78) |
| SBP, mmHg |  |  |  |  |  |  |
| <140 | ref | ref | ref | ref | ref | ref |
| 140-<160 | 1.38 (1.13,1.68) | 1.30 (1.05,1.60) | 1.29 (1.05,1.60) | 1.21 (0.97,1.52) | 2.18 (1.20,3.96) | 2.08 (1.09,3.95) |
| ≥160 | 2.22 (1.80,2.73) | 1.86 (1.49,2.32) | 2.14 (1.72,2.67) | 1.78 (1.40,2.26) | 2.97 (1.63,5.40) | 2.69 (1.41,5.15) |
| BMI, kg/m^2^ |  |  |  |  |  |  |
| <28 | ref | ref | ref | ref | ref | ref |
| ≥28 | 1.17 (0.99,1.39) | 0.99 (0.82,1.19) | 1.16 (0.96,1.4) | 0.98 (0.8,1.21) | 1.21 (0.79,1.86) | 0.95 (0.59,1.54) |
| Smoking status |  |  |  |  |  |  |
| Never | ref | ref | ref | ref | ref | ref |
| Ever | 1.24 (0.99,1.55) | 1.27 (1,1.6) | 1.36 (1.07,1.73) | 1.36 (1.05,1.75) | 0.68 (0.37,1.26) | 0.76 (0.39,1.51) |
| Diabetes |  |  |  |  |  |  |
| No | ref | ref | ref | ref | ref | ref |
| Yes | 1.82 (1.51,2.21) | 1.61 (1.32,1.97) | 2 (1.63,2.46) | 1.75 (1.41,2.17) | 0.96 (0.57,1.64) | 1.05 (0.57,1.91) |
| TC, mmol/L |  |  |  |  |  |  |
| <5.2 | ref | ref | ref | ref | ref | ref |
| ≥5.2 | 0.96 (0.81,1.14) | 0.97 (0.8,1.17) | 0.97 (0.81,1.17) | 0.98 (0.8,1.2) | 0.89 (0.56,1.43) | 0.94 (0.53,1.66) |
| TG, mmol/L |  |  |  |  |  |  |
| <1.7 | ref | ref | ref | ref | ref | ref |
| ≥1.7 | 1.48 (1.23,1.77) | 1.27 (1.03,1.56) | 1.58 (1.29,1.92) | 1.34 (1.07,1.68) | 1.00 (0.62,1.62) | 0.86 (0.48,1.51) |
| HDL, mmol/L |  |  |  |  |  |  |
| male≥1.03/female≥1.3 | ref | ref | ref | ref | ref | ref |
| male<1.03/female<1.3 | 1.37 (1.06,1.76) | 1.18 (0.89,1.57) | 1.47 (1.12,1.93) | 1.21 (0.89,1.65) | 0.81 (0.39,1.69) | 0.92 (0.38,2.25) |
| Center: Lianyungang |  |  |  |  |  |  |
| tHcy, μmol/L |  |  |  |  |  |  |
| <10 | ref | ref | ref | ref | ref | ref |
| 10-15 | 1.13 (0.97,1.32) | 1.13 (0.96,1.32) | 1.10 (0.94,1.30) | 1.11 (0.93,1.31) | 1.29 (0.80,2.07) | 1.32 (0.77,2.26) |
| ≥15 | 1.57 (1.30,1.91) | 1.49 (1.21,1.83) | 1.51 (1.23,1.86) | 1.45 (1.17,1.81) | 2.11 (1.18,3.78) | 1.60 (0.80,3.17) |
| SBP, mmHg |  |  |  |  |  |  |
| <140 | ref | ref | ref | ref | ref | ref |
| 140-160 | 1.47 (1.25,1.72) | 1.42 (1.21,1.67) | 1.40 (1.19,1.65) | 1.36 (1.14,1.61) | 2.64 (1.48,4.71) | 2.59 (1.40, 4.80) |
| ≥160 | 1.98 (1.64,2.40) | 1.82 (1.49,2.22) | 1.78 (1.45,2.19) | 1.64 (1.32,2.03) | 4.99 (2.59,9.61) | 5.10 (2.47,10.53) |
| BMI, kg/m^2^ |  |  |  |  |  |  |
| <28 | ref | ref | ref | ref | ref | ref |
| ≥28 | 1.18 (1.02,1.36) | 1.03 (0.88,1.2) | 1.21 (1.04,1.41) | 1.06 (0.9,1.25) | 1.18 (0.77,1.79) | 0.83 (0.5,1.37) |
| Smoking status |  |  |  |  |  |  |
| Never | ref | ref | ref | ref | ref | ref |
| Ever | 1.05 (0.89,1.24) | 1.12 (0.94,1.33) | 0.99 (0.82,1.19) | 1.08 (0.89,1.3) | 1.59 (1.00,2.52) | 1.11 (0.65,1.91) |
| Diabetes |  |  |  |  |  |  |
| No | ref | ref | ref | ref | ref | ref |
| Yes | 1.43 (1.24,1.66) | 1.34 (1.15,1.57) | 1.54 (1.32,1.81) | 1.46 (1.23,1.72) | 1 .00(0.64,1.57) | 0.76 (0.45,1.29) |
| TC, mmol/L |  |  |  |  |  |  |
| <5.2 | ref | ref | ref | ref | ref | ref |
| ≥5.2 | 1.03 (0.91,1.16) | 1.00 (0.87,1.14) | 0.97 (0.85,1.11) | 0.95 (0.82,1.1) | 1.57 (1.07,2.29) | 1.45 (0.93,2.26) |
| TG, mmol/L |  |  |  |  |  |  |
| <1.7 | ref | ref | ref | ref | ref | ref |
| ≥1.7 | 1.21 (1.06,1.38) | 1.05 (0.91,1.21) | 1.23 (1.07,1.41) | 1.06 (0.91,1.24) | 1.04 (0.71,1.52) | 0.97 (0.6,1.57) |
| HDL, mmol/L |  |  |  |  |  |  |
| male≥1.03/female≥1.3 | ref | ref | ref | ref | ref | ref |
| male<1.03/female<1.3 | 1.10 (0.94,1.29) | 1.05 (0.88,1.25) | 1.15 (0.97,1.36) | 1.08 (0.89,1.3) | 0.74 (0.46,1.2) | 0.73 (0.4,1.33) |

Adjusted for tHcy, SBP, BMI, smoking, diabetes, eGFR, triglyceride, HDL, physical intensity of job, antihypertensive drugs, CHD.

**Appendix Table 4.** Stratified analysis of the associations of tHcy with the risk of first stroke, ischemic and hemorrhagic stroke.

|  | Total | | | | P for interaction | Ischemic stroke | | | | P for interaction | Hemorrhagic stroke | | | | P for interaction |
| --- | --- | --- | --- | --- | --- | --- | --- | --- | --- | --- | --- | --- | --- | --- | --- |
|  | N | Events (%) | Adjusted | P |  | N | Events (%) | Adjusted | P |  | N | Events (%) | Adjusted | P |  |
| SBP, mmHg |  |  |  |  | 0.691 |  |  |  |  | 0.243 |  |  |  |  | 0.182 |
| <140 | 2043 | 873 (42.7) | 1.07 (1,1.14) | 0.065 |  | 1817 | 793 (43.6) | 1.06 (0.98,1.14) | 0.13 |  | 212 | 74 (34.9) | 1.13 (0.93,1.36) | 0.218 |  |
| 140-<160 | 2980 | 1482 (49.7) | 1.03 (0.98,1.09) | 0.263 |  | 2596 | 1291 (49.7) | 1.01 (0.96,1.07) | 0.596 |  | 356 | 179 (50.3) | 1.28 (1.03,1.59) | 0.028 |  |
| ≥160 | 2043 | 1178 (57.7) | 1.08 (1.01,1.16) | 0.027 |  | 1727 | 986 (57.1) | 1.12 (1.03,1.21) | 0.008 |  | 284 | 173 (60.9) | 1.01 (0.87,1.18) | 0.89 |  |
| Age |  |  |  |  | 0.362 |  |  |  |  | 0.702 |  |  |  |  | 0.481 |
| <65 | 2625 | 1312 (50) | 1.04 (0.97,1.11) | 0.329 |  | 2236 | 1118 (50) | 1.05 (0.98,1.13) | 0.172 |  | 349 | 174 (49.9) | 0.99 (0.77,1.28) | 0.963 |  |
| ≥65 | 4441 | 2221 (50) | 1.06 (1.02,1.11) | 0.006 |  | 3904 | 1952 (50) | 1.05 (1.01,1.1) | 0.029 |  | 503 | 252 (50.1) | 1.14 (1,1.3) | 0.057 |  |
| Sex |  |  |  |  | 0.332 |  |  |  |  | 0.107 |  |  |  |  | 0.227 |
| Male | 3160 | 1580 (50) | 1.04 (1,1.09) | 0.035 |  | 2730 | 1365 (50) | 1.03 (0.99,1.08) | 0.123 |  | 406 | 203 (50) | 1.13 (0.99,1.28) | 0.08 |  |
| Female | 3906 | 1953 (50) | 1.1 (1.02,1.19) | 0.016 |  | 3410 | 1705 (50) | 1.12 (1.03,1.22) | 0.008 |  | 446 | 223 (50) | 1.01 (0.81,1.27) | 0.904 |  |
| BMI, kg/m^2^ |  |  |  |  | 0.46 |  |  |  |  | 0.391 |  |  |  |  | 0.804 |
| <28 | 5147 | 2519 (48.9) | 1.06 (1.02,1.1) | 0.004 |  | 4495 | 2198 (48.9) | 1.05 (1.01,1.1) | 0.016 |  | 600 | 292 (48.7) | 1.14 (1,1.31) | 0.048 |  |
| ≥28 | 1889 | 998 (52.8) | 1.04 (0.95,1.13) | 0.41 |  | 1618 | 858 (53) | 1.05 (0.95,1.15) | 0.335 |  | 249 | 132 (53) | 0.99 (0.78,1.26) | 0.936 |  |
| Smoking status | |  |  |  | 0.217 |  |  |  |  | 0.112 |  |  |  |  | 0.42 |
| Never | 5075 | 2514 (49.5) | 1.08 (1.02,1.14) | 0.005 |  | 4421 | 2191 (49.6) | 1.09 (1.02,1.15) | 0.005 |  | 593 | 292 (49.2) | 1.07 (0.93,1.23) | 0.32 |  |
| Ever | 1991 | 1019 (51.2) | 1.03 (0.98,1.09) | 0.205 |  | 1719 | 879 (51.1) | 1.02 (0.97,1.08) | 0.4 |  | 259 | 134 (51.7) | 1.11 (0.94,1.32) | 0.206 |  |
| Diabetes |  |  |  |  | 0.02 |  |  |  |  | 0.08 |  |  |  |  | 0.007 |
| No | 5548 | 2640 (47.6) | 1.08 (1.03,1.12) | <0.001 |  | 4795 | 2260 (47.1) | 1.07 (1.02,1.11) | 0.005 |  | 697 | 349 (50.1) | 1.25 (1.07,1.47) | 0.006 |  |
| Yes | 1518 | 893 (58.8) | 1 (0.94,1.06) | 0.964 |  | 1345 | 810 (60.2) | 1.02 (0.95,1.09) | 0.582 |  | 155 | 77 (49.7) | 0.69 (0.47,0.99) | 0.046 |  |
| TG, mmol/L | |  |  |  | 0.926 |  |  |  |  | 0.883 |  |  |  |  | 0.933 |
| <1.7 | 4994 | 2407 (48.2) | 1.06 (1.02,1.1) | 0.008 |  | 4338 | 2082 (48) | 1.05 (1.01,1.1) | 0.024 |  | 608 | 303 (49.8) | 1.12 (0.98,1.28) | 0.087 |  |
| ≥1.7 | 2072 | 1126 (54.3) | 1.04 (0.97,1.12) | 0.228 |  | 1802 | 988 (54.8) | 1.05 (0.97,1.13) | 0.216 |  | 244 | 123 (50.4) | 1 (0.8,1.26) | 0.994 |  |
| HDL, mmol/L | |  |  |  | 0.022 |  |  |  |  | 0.007 |  |  |  |  | 0.028 |
| male≥1.03/female≥1.3 | 5968 | 2950 (49.4) | 1.07 (1.03,1.12) | <0.001 |  | 5178 | 2550 (49.2) | 1.08 (1.03,1.13) | <0.001 |  | 731 | 372 (50.9) | 1.08 (0.97,1.2) | 0.159 |  |
| male<1.03/female<1.3 | 1098 | 583 (53.1) | 0.98 (0.92,1.05) | 0.593 |  | 962 | 520 (54.1) | 0.97 (0.91,1.04) | 0.437 |  | 121 | 54 (44.6) | 1.91 (1.07,3.43) | 0.03 |  |
| LDL, mmol/L | |  |  |  | 0.039 |  |  |  |  | 0.077 |  |  |  |  | 0.523 |
| <3.4 | 4165 | 2027 (48.7) | 1.03 (0.99,1.07) | 0.169 |  | 3597 | 1759 (48.9) | 1.03 (0.98,1.07) | 0.258 |  | 516 | 244 (47.3) | 1.08 (0.96,1.22) | 0.192 |  |
| ≥3.4 | 2901 | 1506 (51.9) | 1.12 (1.04,1.2) | 0.002 |  | 2543 | 1311 (51.6) | 1.11 (1.03,1.2) | 0.005 |  | 336 | 182 (54.2) | 1.21 (0.97,1.51) | 0.091 |  |
| Physical intensity of job | | |  |  |  |  |  |  |  | 0.305 |  |  |  |  | 0.793 |
| Heavy | 2595 | 1232 (47.5) | 1.06 (1,1.13) | 0.057 |  | 2233 | 1057 (47.3) | 1.06 (0.99,1.13) | 0.116 |  | 320 | 154 (48.1) | 1.11 (0.94,1.31) | 0.217 |  |
| Light | 4471 | 2301 (51.5) | 1.05 (1.01,1.1) | 0.022 |  | 3907 | 2013 (51.5) | 1.05 (1,1.1) | 0.037 |  | 532 | 272 (51.1) | 1.11 (0.96,1.29) | 0.153 |  |
| Coronary Heart Disease | |  |  |  | 0.874 |  |  |  |  | 0.847 |  |  |  |  | 0.803 |
| No | 6420 | 3168 (49.3) | 1.05 (1.01,1.09) | 0.007 |  | 5582 | 2751 (49.3) | 1.05 (1.01,1.09) | 0.018 |  | 772 | 386 (50) | 1.1 (0.99,1.22) | 0.087 |  |
| Yes | 646 | 365 (56.5) | 1.07 (0.94,1.23) | 0.323 |  | 558 | 319 (57.2) | 1.06 (0.92,1.21) | 0.419 |  | 80 | 40 (50) | 1.02 (0.5,2.08) | 0.961 |  |

Adjusted for SBP, BMI, smoking, diabetes, eGFR, triglyceride, HDL, physical intensity of job, antihypertensive drugs, CHD.

**Appendix Table 5.** Studies reporting population attributable risks for first stroke.

| Study | Year | Design | Outcome | Variables | Population attributable risk of hypertension |
| --- | --- | --- | --- | --- | --- |
| Ezzati [37] | 2003 | in developing countries, multiple center | first stroke | hypertension, body mass index (BMI), high total cholesterol, fruit and vegetable, physical inactivity, smoker, alcohol | 58% |
| Hankey [38] | 2006 | Rochester, Minn of USA; population- based case-control | first ischemic stroke | hypertension, transient ischemic attack, smoker, ischemic heart disease, atrial fibrillation (AF), diabetes, mitral valve disease | 26% |
| O'Donnell [39] | 2010 | in 14 countries; in 2007-2010, hospital- based, age (± 5) and sex matched case-control | first stroke (hemorrhagic or ischemic） | hypertension, smoker, waist-to-hip ratio, diet, physical activity, diabetes, alcohol, psychosocial factors, cardiac causes, ApoB/A1 ratio | NA |
| Mallmann [40] | 2012 | Brazil; in 2009-2010; age ≥ 45 yrs; hospital-based, age (± 2) and sex matched case-control | first ischemic stroke | hypertension, left ventricular hypertrophy, physical inactivity, high-density lipoprotein, smoking, carotid bruit, diabetes, alcohol abuse, AF | 84.9% |
| Bos [41] | 2014 | Netherlands; enrolled in 1990; age ≥ 55 yrs; prospective population-based cohort study | first stroke (hemorrhagic or ischemic) | hypertension, smoker, diabetes, AF, coronary disease, BMI | 36% |
| Park [42] | 2015 | Korean; in 2008; age range to 19-90 yrs; age (± 3) and sex matched case-control | first ischemic stroke | hypertension, diabetes, smoking, hypercholesterolemia, stroke history, coronary heart disease, BMI. | male (  < 45 yrs, 28.45%; 46-65 yrs, 22.66%;  ≥ 66 yrs, 23.67%); female (  < 45 yrs, 5.39%; 46-65 yrs, 22.69%;  ≥ 66 yrs, 23.40%) |
| Malsch [43] | 2018 | German; since 2010; age ≥ 18 yrs; prospective hospital-based cohort | poor outcome one year after first ischemic stroke | age, education, physical disability, diabetes, the national institutes of health stroke scale | none |
| Owolabi [44] | 2018 | Ghana and Nigeria; in 2011; age ≥ 18 yrs; multicenter hospital-based matched case-control | first stroke (ischemic, hemorrhagic) | age, education, monthly income, hypertension, diabetes, waist-to-hip ratio, dyslipidemia, cardiac disease, physical inactivity, smoker, stress, family history of cardiovascular diseases (CVD), salt, vegetable consumption, sugar, meat | first stroke, 90.8%;  ischemic, 86.6%; hemorrhagic, 96.6% |
| Akpalu [11] | 2019 | in West Africa; in 2011; age ≥ 18 yrs; multicenter, age and sex matched case-control | first stroke (ischemic, hemorrhagic) | age, education, monthly income, hypertension, diabetes, waist-to-hip ratio, dyslipidemia, cardiac disease, physical inactivity, smoker, stress, family history of CVD, salt, vegetable consumption, sugar, meat | male, 89.7%;  female, 92.7% |
| Dong [45] | 2020 | in 14 provinces of China; in 2016, age ≥ 40 yrs, multi-center, hospital-based cross-sectional study | first stroke (ischemic, hemorrhagic) | hypertension, diabetes, dyslipidemia, heart disease, smoker, overweight, physical inactivity, family history of stroke | national, 61.5%; Eastern, 64%; Central, 67.7%; Western, 50.5% |
| Yi [46] | 2020 | in West China, age ≥ 40 yrs, multi-center, community-based cross-sectional study | first stroke (ischemic, hemorrhagic) | hypertension, obesity, smoke, physical inactivity, diabetes, dyslipidemia, AF, family history | first stroke, 23.6%; ischemic, 20.2%; hemorrhagic, 15.3% |
| This study | 2021 | in rural China, in 2016-2018; age ≥ 35 yrs, prospective, age and sex matched nested case-control study | first stroke (ischemic, hemorrhagic) | SBP, Hcy, physical intensity of job, diabetes, smoker, coronary heart disease, BMI, triglycerides, high-density lipoprotein, low-density lipoprotein | population attributable risk of SBP:  first stroke, 26%; ischemic, 22%; hemorrhagic, 51% |

[37] Ezzati M, Hoorn SV, Rodgers A, Lopez AD, Mathers CD, Murray CJ, *et al*. Estimates of global and regional potential health gains from reducing multiple major risk factors. *Lancet* (2003) 362:271-80. doi: 10.1016/s0140-6736(03)13968-2

[38] Hankey GJ. Potential new risk factors for ischemic stroke: what is their potential?. *Stroke.* (2006) 37:2181-8. doi: 10.1161/01.STR.0000229883.72010.e4

[39] O'Donnell MJ, Xavier D, Liu L, Zhang H, Chin SL, Rao-Melacini P, *et al*. Risk factors for ischaemic and intracerebral haemorrhagic stroke in 22 countries (the INTERSTROKE study): a case-control study. *Lancet* (2010) 376:112-23. doi: 10.1016/S0140-6736(10)60834-3

[40] Mallmann AB, Fuchs SC, Gus M, Fuchs FD, Moreira LB. Population-attributable risks for ischemic stroke in a community in South Brazil: a case-control study. *PLoS One* (2012) 7:e35680. doi: 10.1371/journal.pone.0035680

[41] Bos MJ, Koudstaal PJ, Hofman A, Ikram MA. Modifiable etiological factors and the burden of stroke from the Rotterdam study: a population-based cohort study. *PLoS Med* (2014) 11:e1001634. doi: 10.1371/journal.pmed.1001634

[42] Park TH, Ko Y, Lee SJ, Lee KB, Lee J, Han MK, *et al*. Identifying Target Risk Factors Using Population Attributable Risks of Ischemic Stroke by Age and Sex. *J Stroke* (2015) 17:302-11. doi: 10.5853/jos.2015.17.3.302.

[43] Malsch C, Liman T, Wiedmann S, Siegerink B, Georgakis MK, Tiedt S, *et al*. Outcome after stroke attributable to baseline factors-The PROSpective Cohort with Incident Stroke (PROSCIS). *PLoS One* (2018) 13: e0204285. doi: 10.1371/journal.pone.0204285

[44] Owolabi MO, Sarfo F, Akinyemi R, Gebregziabher M, Akpa O, Akpalu A, *et al*. Dominant modifiable risk factors for stroke in Ghana and Nigeria (SIREN): a case-control study. *Lancet Glob Health* (2018) 6: e436-46. doi: 10.1016/S2214-109X(18)30002-0

[45] Dong S, Fang J, Li Y, Ma M, Hong Y, He L. The population attributable risk and clustering of stroke risk factors in different economical regions of China. *Medicine (Baltimore)* 2020;99: e19689. doi: 10.1097/MD.0000000000019689

[46] Yi X, Luo H, Zhou J, Yu M, Chen X, Tan L, *et al*. Prevalence of stroke and stroke related risk factors: a population based cross sectional survey in southwestern China. *BMC Neurol* (2020) 20:5. doi: 10.1186/s12883-019-1592-z
